# Impact of the adjunctive use criteria for intravascular ultrasound-guided percutaneous coronary intervention and clinical outcomes

**DOI:** 10.1038/s41598-022-27250-3

**Published:** 2023-01-13

**Authors:** Pannipa Suwannasom, Ply Chichareon, Worawut Roongsangmanoon, Artit Thongtanomkul, Anuchit Wongpen, Muenpetch Muenkaew, Anek Kanoksilp, Mann Chandavimol, Srun Kuanprasert, Ammarin Thakkinstian, Suphot Srimahachota, Nakarin Sansanayudh

**Affiliations:** 1 Division of Cardiology, Department of Internal Medicine, Faculty of Medicine, Chiang Mai University, Chiang Mai, Thailand; 2 Faculty of Medicine, Songklanakarind Hospital, Prince of Songkla University, Songkla, Thailand; 3 Faculty of Medicine, HRH Princess MahaChakri Sirindhorn Medical Center, Srinakharinwirot University, Nakhon Nayok, Thailand; 4 Maharaj Nakorn Si Thammarat Hospital, Nakorn Si Thammarat, Thailand; 5 Udonthani Hospital, Udonthani, Thailand; 6 Faculty of Medicine, Thammasat University Hospital, Thammasat University, Bangkok, Thailand; 7 Central Chest Institute of Thailand, Nonthaburi, Thailand; 8 Division of Cardiology, Department of Medicine, Faculty of Medicine Ramathibodi Hospital, Mahidol University, Bangkok, Thailand; 9 Department of Clinical Epidemiology and Biostatistics, Faculty of Medicine, Ramathibodi Hospital, Mahidol University, Bangkok, Thailand; 10 Division of Cardiovascular Diseases, Department of Medicine, King Chulalongkorn Memorial Hospital, Bangkok, Thailand; 11 Phramongkutklao College of Medicine, 315 Ratchawithi Rd, Khwaeng Thung Phaya Thai, Bangkok, 10400 Thailand

**Keywords:** Cardiology, Health care

## Abstract

The impact of the adherence to the adjunctive use criteria (AUC) for intravascular ultrasound (IVUS) guided percutaneous coronary intervention (PCI) and clinical outcomes in low IVUS volume countries are limited. The current study compared the procedural success and complication rates between used and not used IVUS catheter in the patients who were met (C +) and were not met (C−) the AUC for IVUS-guided PCI. From June 2018 through June 2019, a total of 21,066 patients were included in the Thai PCI registry. Among the study population, 15,966 patients (75.8%) have met the IVUS-AUC. The IVUS-guided PCI rates were 14.5% and 11.3% in the C + and C − groups, respectively. After adjusting for covariables by propensity model, IVUS-guided PCI was identified as an independent predictor of the procedural success rate regardless of whether the AUC were met with the relative risk [RR (95% confidence interval (CI)] of 1.033(1.026–1.040) and 1.012(1.002–1.021) in C + and C− groups, respectively. IVUS-guided PCI increased the procedural complication risks in both groups but were not significant with corresponding RRs of 1.171(0.915–1.426) and 1.693(0.959–2.426). Procedural success was achieved with IVUS-guided PCI regardless of whether the AUC were met. IVUS-guided PCI did not lead to an increase in procedural complications.

## Introduction

Angiography-guided percutaneous coronary intervention (PCI) has been used in clinical practice for many decades. In the modern era, the benefits of intracoronary imaging in reducing cardiac death, target vessel myocardial infarction (MI), and target vessel revascularization have been repeatedly demonstrated in randomized controlled trials^1,2^ and meta-analyses^3–6^. However, the penetrance of intracoronary imaging-guided PCI in real-world practice remains low. The use of intracoronary imaging-guided PCI, especially intravascular ultrasound (IVUS), is most common in Japan (75.0–84.8%)^7,8^, followed by Korea (27.5–27.9%)^9,10^ and Germany (16.2%)^11^, while the USA^12,13^ and Italy^14^ have rates of around 5%. Despite the huge disparities in IVUS utilization among countries, associations between IVUS-guided PCI and low rates of long-term mortality^8^ and repeat revascularization^7,10,12,13^ have been reported consistently.

A recent expert consensus document from the European Association of Percutaneous Cardiovascular Interventions (EAPCI)^15^ recommended consideration of the adjunctive use of intravascular imaging for PCI guidance in patients with long lesions, chronic total occlusion (CTO), acute coronary syndrome (ACS), left main disease, two-stent bifurcation, implantation of bioresorbable scaffolds, or renal dysfunction. The consensus was derived from the published data that prespecified the used of IVUS-guided PCI in complex lesions^10,16–18^ or patients with complex clinical setting such as renal dysfunction or ACS^19–21^. In addition, some IVUS-guided PCI trials had pre-defined criteria for stent optimization in IVUS-guided PCI arm^22,23^. Recently, patients-level analysis from 2 randomized trials^6^ (IVUS-XPL [Impact of Intravascular Ultrasound Guidance on the Outcomes of Xience Prime Stents in Long Lesions]^17^ and ULTIMATE [Intravascular Ultrasound Guided Drug Eluting Stents Implantation in All-Comers Coronary Lesions])^1^ showed that IVUS-guided PCI in patients with long lesions with stent length ≥ 28 mm improved 3-year patients cardiac survival^6^. However, the impact of the adherence to the adjunctive use criteria (AUC) for IVUS on PCI guidance and clinical outcomes remains controversial. The present study was performed to investigate the IVUS procedural success and complication rates according to adherence to the AUC of IVUS in real-world clinical practice.

## Methods

### Study population

The Thai PCI Registry is a prospective, multi-centre study initiative project of the Cardiac Intervention Association of Thailand. The study design and protocol of the Thai PCI registry were described previously^24,25^. Briefly, the study was conducted at 39 centres in Thailand. All consecutive patients undergoing PCI who were aged 18 years or older were enrolled. Written informed consent was obtained from all patients. The details regarding the patient characteristics, procedural information, equipment, and outcomes of PCI were prospectively collected in each participating centre and recorded in electronic case record forms.

The Thai PCI registry was conducted in accordance with the Declaration of Helsinki and the Ethical Guidelines for Human Study. The protocol was approved by the Central Research Ethics Committee of Thailand (approval no. COA-CREC 006/2018) and the local ethics committee if required.

### Adjunctive use of IVUS criteria

The adjunctive use of IVUS criteria were modified from the EAPCI expert consensus document^15^ as follows: long lesions (lesion length > 30 mm), CTO, left main disease, true bifurcation lesion with Medina classification 1,1,1/ 0,1,1/ 1,0,1 and number of stent use of 2 or more, in-stent restenosis, stent thrombosis, renal dysfunction (glomerular filtration rate [GFR] < 60 ml/min), and ACS receiving PCI within 1 week after onset. Patients treated with balloon angioplasty or receiving PCI under optical coherence tomography (OCT) guidance were excluded from the analysis.

Patients who did and did not meet at least one of the adjunctive use criteria for IVUS were classified into the C + and C − groups, respectively. The patients in each group were further divided into the IVUS-guided PCI (I +) and angio-guided PCI (I-) groups according to the actual utilization of IVUS during the procedure. In patients with multi-vessel PCI, the lesion treated under IVUS guidance was selected as the representative lesion. In cases in which IVUS-guided PCI was performed in more than one lesion, the representative lesions were prioritized as follows: left main, CTO, bifurcation, long lesion, in-stent restenosis, and stent thrombosis.

### Study endpoints

The primary endpoint was the procedural success rate, defined as achievement of < 30% residual stenosis of the target lesion as assessed by visual estimation and without in-hospital major adverse cardiac events (death, MI, target lesion revascularization, or stent thrombosis). The secondary endpoints were the procedural complication rate and 1-year all-cause mortality rate. Clinical follow-up was performed via hospital visits or telephone calls at 6 and 12 months.

### Statistical analysis

All continuous variables are presented as the mean ± standard deviation (SD) or median and interquartile range as appropriate. The categorical variables are reported as frequencies and percentages. In the primary analysis, IVUS-guided (I +) or angio-guided PCI(I−) was evaluated according to the C+ or C− group. Clinical and procedural characteristics are summarized according to whether the adjunctive use criteria were met. Comparisons between groups were performed using the χ^2^ test or Fisher’s exact test for categorical variables and Student’s *t* test for continuous variables. A univariate and multivariate logistics regression were performed to evaluate the factor associated with procedural success, procedural complication, in-hospital mortality, and 1-year mortality rates in patients who met and unmet criteria.

Effect of IVUS guided-PCI on clinical outcomes was assessed using a propensity analysis by an inverse probability weighting and regression adjustment (IPWRA) stratify by C + /C− groups as follows: First, a propensity model was constructed applying a logit equation by fitting IVUS on variables might be associated with IVUS application and also the clinical outcome of interests^26,27^ including demographic data [i.e., age, sex, body mass index (BMI)], Clinical presentations (i.e., ST-elevation myocardial infarction, non ST-elevation myocardial infarction, chronic coronary syndrome), chronic total occlusion, previously treated lesions, co-morbidities (i.e., hypertension, diabetes, dyslipidemia, peripheral artery disease, prior-myocardial infarction, prior coronary artery bypass graft surgery, known coronary artery disease, chronic kidney disease, cerebrovascular disease), femoral access site, lesion complexity, total volume contrast, plaque modification, and bifurcation. Only significant variables were kept in the final propensity model. Balancing of these significant variables between IVUS and non-IVUS groups was checked. The propensity model was well specified if absolute weighted standardized mean differences of these significant variables did not exceed 0.2^28^. Second, the outcome model was constructed weighted by propensity score estimated from the first step. A potential outcome mean (i.e., a risk) of outcome occurrence was estimated by IVUS groups. Average treatment effect or risk difference (RD) was then estimated by subtracting risks between IVUS-guided and angio-guided groups. Furthermore, relative risk (RR) was finally estimated by diving risk in IVUS-guided and angio-guided group.

All reported *P*-values are two-sided, and *P* < 0.05 was taken to indicate statistical significance. Statistical analyses were performed using STATA version 17 (Stata Corp., College Station, TX, USA).

## Results

### Baseline characteristics

**Figure 1:**
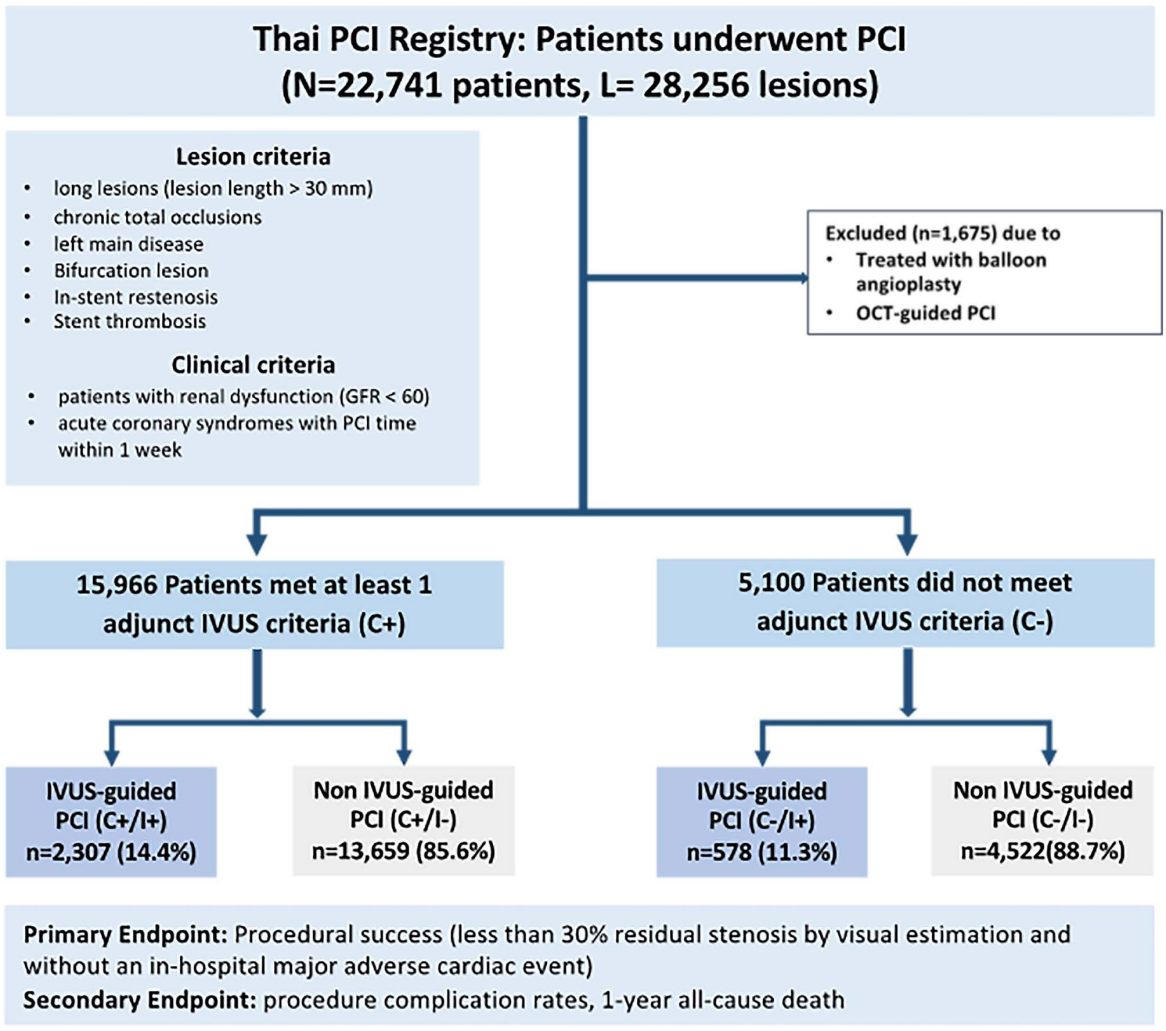
Patient flow chart. Abbreviations: C + , met criteria; C−, unmet criteria; GFR, glomerular filtration rate; I + , IVUS-guided PCI, I−, angio-guided PCI; IVUS, intravascular ultrasound; MI, myocardial infarction; OCT, optical coherence tomography; PCI, percutaneous coronary intervention.

A total of 22,741 patients were enrolled in the Thai PCI registry with mean age of 64.2 years and percent male was 64.0%. Of these patients, 1,675 who were treated with balloon angioplasty or OCT for PCI guidance were excluded from the analysis (mean age of 64.7 years, male 66.4%). Finally, 21,066 patients were included in the study, of whom 15,966 (75.8%) met at least one adjunctive use criteria for IVUS (C + group), and 5,100 (24.2%) did not meet any of these criteria (C − group). IVUS-guided PCI (I + group) and angio-guided PCI (I- group) were performed in 14.5% and 85.6% of patients in the C + group and in 11.3% and 88.7% in the C − group, respectively. The study flow chart is presented in Fig. 1.

The baseline characteristics are shown in Table 1. The rates of hypertension, hyperlipidaemia, peripheral artery disease, and prior history of revascularization with either PCI or coronary artery bypass graft surgery were significantly higher in the patients group C + /I + than the group C + /I−. Conversely, patients presenting with ACS or cardiogenic shock were more likely to be treated with angio-guided PCI than IVUS-guided PCI. Potent P2Y12 inhibitors, such as prasugrel and ticagrelor, were prescribed at discharge more commonly in patients treated with IVUS-guided PCI.

**Table 1:** Baseline patient characteristics.

Characteristics	C + group			C − group		
	IVUS-guided PCI (I +)	Angio-guided PCI (I-)	*P*-value	IVUS-guided PCI (I +)	Angio-guided PCI (I-)	*P*-value
	n = 2,307	n = 13,659		n = 578	n = 4,522	
Age, years	64.1 (11.9)	64.8 (11.9)	0.006	62.3 (10.8)	62.3 (10.7)	0.990
Male	1581 (68.5)	9371 (68.6)		399 (69.0)	3238 (71.6)	
Diabetes mellitus	1048 (45.4)	6294 (46.1)	0.560	210 (36.3)	1628 (36.0)	0.880
CKD	915 (39.7)	5846 (42.8)	0.005	0 (0.0)	0 (0.0)	–
On dialysis	119 (5.2)	631 (4.6)	0.260	0 (0.0)	0 (0.0)	–
Hypertension	1619 (70.2)	8933 (65.4)	< 0.001	414 (71.6)	3155 (69.8)	0.360
Dyslipidemia	1535 (66.5)	8485 (62.1)	< 0.001	435 (75.3)	3288 (72.7)	0.190
Peripheral arterial disease	60 (2.6)	194 (1.4)	< 0.001	9 (1.6)	69 (1.5)	0.950
Prior PCI	905 (39.2)	2959 (21.7)	< 0.001	269 (46.5)	1824 (40.3)	0.004
Prior CABG	49 (2.1)	201 (1.5)	0.02	9 (1.6)	49 (1.1)	0.310
Prior MI	709 (30.7)	2561 (18.7)	< 0.001	215 (37.2)	1390 (30.7)	0.002
Cardiogenic shock at start of PCI	166 (7.2)	1388 (10.2)	< 0.001	2 (0.3)	37 (0.8)	0.220
**CAD presentation: /indication**			< 0.001			< 0.001
STEMI	418 (18.1)	5271 (38.6)		3 (0.5)	145 (3.2)	
NSTEMI	643 (27.9)	4301 (31.5)		115 (19.9)	1290 (28.5)	
Stable CAD	1246 (54.0)	4087 (29.9)		460 (79.6)	3087 (68.3)	
LVEF, %	50.8 (16.1)	50.2 (15.2)	0.210	55.3 (15.7)	55.5 (15.3)	0.780
**Discharge medication**						
Aspirin	2227 (98.1)	12,998 (97.1)	0.006	573 (99.3)	4456 (98.7)	0.210
Clopidogrel	1726 (76.1)	10,722 (80.1)	< 0.001	444 (76.9)	3855 (85.4)	< 0.001
Prasugrel	107 (4.7)	292 (2.2)	< 0.001	31 (5.4)	151 (3.3)	0.013
Ticagrelor	373 (16.4)	1,945 (14.5)	0.018	99 (17.2)	452 (10.0)	< 0.001

Values are expressed as *n* (%) or mean ± SD.

Abbreviations: C + , met criteria; C−, unmet criteria; CABG, coronary artery bypass graft; CAD, coronary artery disease; CKD, chronic kidney disease; IVUS, intravascular ultrasound; I + , IVUS-guided PCI, I−, angio-guided PCI; LVEF, left ventricular ejection fraction; MI, myocardial infarction; NSTEMI, non-ST segment elevation myocardial infarction; PCI, percutaneous coronary intervention; STEMI, ST segment elevation myocardial infarction.

The underlying medical conditions were comparable between the IVUS-guided and angio-guided PCI groups within the C − group, except for histories of prior PCI and MI, both of which had higher rates in the IVUS-guided group. Again, we found that potent P2Y12 inhibitors were used more frequently in patients treated with IVUS-guided PCI within the C − group.

### Lesion and procedural characteristics

**Figure 2:**
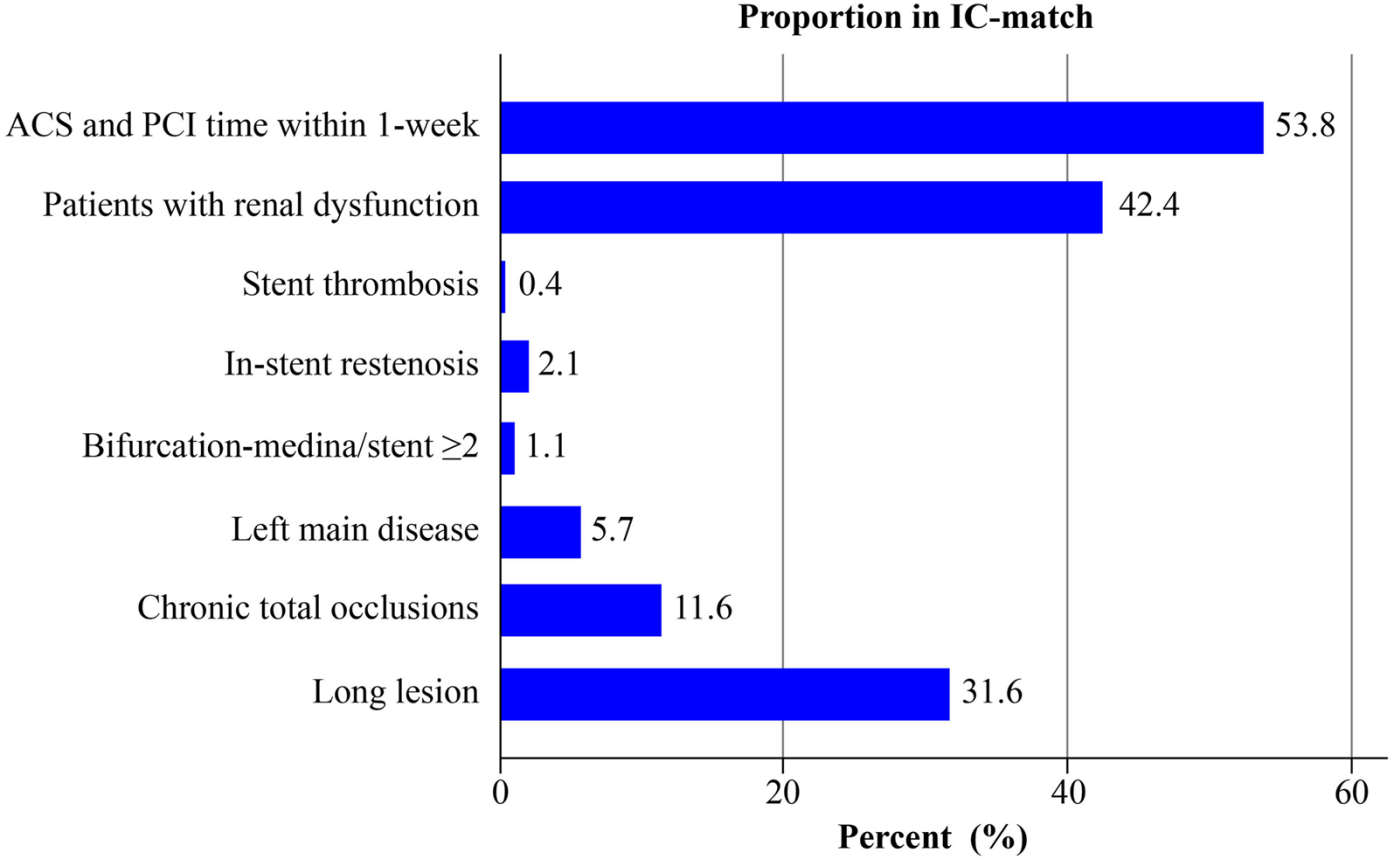
Distribution of the adjunctive use criteria for intravascular ultrasound among the patients who have met the criteria (C + group, n = 15,966). Abbreviations: ACS, acute coronary syndrome; IC, intravascular ultrasound adjunctive use criteria; PCI, percutaneous coronary intervention. Bifurcation lesions that were included in the C + group were true bifurcation lesion with Medina classification 1,1,1/ 0,1,1/ 1,0,1 and number of stent use of 2 or more.

Table 2 summarizes the lesion and procedural characteristics of the patients according to the adjunctive use criteria for IVUS. The proportion of lesion class B2 or C was significantly higher in the IVUS-guided PCI group than in the angio-guided PCI group regardless of whether the AUC were met (C + /I + 88.8% vs. C + /I- 80.8%, *P* < 0.001; C − /I + : 79.9% vs. C–/I– 67.5%, *P* < 0.001). Of those patients who met the AUC, the percentage of each adjunctive use criterion is shown in Fig. 2. The top three most common lesion and clinical criteria were ACS (53.8%), renal dysfunction (42.4%), and long lesion (31.6%) (Fig. 2).

**Table 2:** Lesion and procedural characteristics.

Characteristics	C + group			C − group		
	IVUS-guided PCI (I +)	Angio-guided PCI (I-)	*P*-value	IVUS-guided PCI (I +)	Angio-guided PCI (I-)	*P*-value
	n = 2,307	n = 13,659		n = 578	n = 4,522	
Lesion complexity*			< 0.001			< 0.001
A or B1	258 (11.2)	2596 (19.2)		115 (20.1)	1462 (32.5)	
B2 or C	2039 (88.8)	10,959 (80.8)		456 (79.9)	3033 (67.5)	
Any bifurcation lesion*	691 (30.1)	1513 (11.2)	< 0.001	134 (23.3)	473 (10.5)	< 0.001
Previously treated lesion*	228 (9.9)	581 (4.3)	< 0.001	33 (5.7)	157 (3.5)	0.007
In-stent restenosis*	92 (4.0)	236 (1.7)	< 0.001	0 (0.0)	0 (0.0)	–
In-stent thrombosis	19 (0.8)	40 (0.3)	< 0.001	0 (0.0)	0 (0.0)	–
Chronic total occlusion	498 (21.6)	1361 (10.0)	< 0.001	0 (0.0)	0 (0.0)	–
**Initial access site**			< 0.001			< 0.001
Radial only	824 (35.7)	6143 (45.0)		181 (31.3)	2262 (50.0)	
Brachial only and other	7 (0.3)	28 (0.2)		2 (0.3)	6 (0.1)	
Femoral only	1380 (59.8)	7236 (53.0)		380 (65.7)	2217 (49.0)	
Combination	96 (4.2)	252 (1.8)		15 (2.6)	37 (0.8)	
Plaque modification*	375 (16.3)	383 (2.8)	< 0.001	95 (16.6)	130 (2.9)	< 0.001
Rotational atherectomy	179 (7.8)	158 (1.2)	< 0.001	45 (7.8)	55 (1.2)	< 0.001
Cutting balloon	49 (2.1)	53 (0.4)	< 0.001	10 (1.7)	23 (0.5)	< 0.001
Fluoroscopy time, min	24.2 (14.6, 37.3)	11.9 (7.2, 19.4)	< 0.001	21.2 (13.4, 30.7)	10.3 (6.4, 17.3)	< 0.001
Total volume of contrast, ml, median (IQR)	130.0 (90.0, 170.0)	100.0 (70.0, 130.0)	< 0.001	130.0 (100.0, 170.0)	90.0 (70.0, 125.0)	< 0.001

*There are missing data, percentage is calculated based on total numbers where data is available.

Values are expressed as *n* (%) or mean ± SD or median (interquartile 1st, 3rd).

Abbreviations*:* C + , met criteria; C−, unmet criteria; IVUS, intravascular ultrasound; I + , IVUS-guided PCI, I−, angio-guided PCI; IQR, interquartile range; PCI, percutaneous coronary intervention.

Regardless of the presence of the AUC, femoral access was used more frequently during IVUS-guided PCI than angio-guided PCI. It was also noted that the frequency of using plaque modification procedures, either rotational atherectomy or cutting balloon, was significantly higher under IVUS guidance. IVUS-guided PCI was also related to greater contrast volume and longer fluoroscopic time compared with angio-guided PCI, these findings were observed in both C + and C− group.

### Procedural success and complication rates

The procedural success and complication rates are summarized in Table 3. Procedural success rates were significantly higher in patients who met criteria and treated under IVUS-guided PCI than the angio-guided PCI (C + /I + : 98.1% vs. C + /I– 95.6%, *P* < 0.001). In contrast, procedural success rates in patients who did not meet criteria were comparable irrespective of the usage of imaging catheter (C − /I + : 98.6% vs. C–/I– 97.6%, *P* = 0.127). The procedures performed under IVUS guidance, versus angiography guidance, have led to a higher rate of procedural complications regardless of whether the AUC were met (C + /I + 6.4% vs. C + /I– 5.3%, *P* = 0.036; C-/I + 6.6% vs. C–/I– 2.7%, respectively, *P* < 0.001) (Table 3). The occurrence of coronary perforation was numerically higher in patients in the C + group who were treated with IVUS-guided PCI than with angio-guided PCI, but the difference was not statistically significant (0.6% vs. 0.4%, respectively, *P* = 0.079). In contrast, the rate of coronary perforation was significantly higher in patients in the C − group who were treated with IVUS-guided PCI than with angio-guided PCI (1.0% vs. 0.2%, respectively, *P* < 0.001). Residual dissection and infection were noted at significant rates in patients treated with IVUS-guided PCI regardless of whether the adjunctive use criteria were met. The rate of the no-reflow phenomenon was significantly higher in patients in the C − group who were treated with IVUS-guided PCI than with angio-guided PCI (1.0% vs. 0.2%, respectively, *P* < 0.001). In the C + group, the in-hospital mortality rate was significantly higher in patients who were treated with angio-guided PCI than with IVUS-guided PCI (3.2% vs. 2.3%, respectively, *P* = 0.016). Similarly, the 1-year mortality rate was significantly higher in patients in the C + group who were treated with angio-guided PCI than with IVUS-guided PCI (12.0% vs. 10.2%, respectively, *P* = 0.013). However, the in-hospital mortality and 1-year mortality rates in the C − group were comparable between patients treated with angio-guided PCI and IVUS-guided PCI.

**Table 3:** Procedural success and complication rates.

Outcome	C + group			C − group		
	IVUS-guided PCI (I +)	Angio-guided PCI (I-)	*P*-value	IVUS-guided PCI (I +)	Angio-guided PCI (I-)	*P*-value
	n = 2,307	n = 13,659		n = 578	n = 4,522	
In hospital outcomes						
**Procedural success**						
Success	2263 (98.1)	13,064 (95.6)	< 0.001	570 (98.6)	4414 (97.6)	0.127
Fail	44 (1.9)	595 (4.4)		8 (1.4)	108 (2.4)	
**Any procedural complication**	147 (6.4)	724 (5.3)	0.036	38 (6.6)	123 (2.7)	< 0.001
Perforation	14 (0.6)	49 (0.4)	0.079	6 (1.0)	9 (0.2)	< 0.001
Residual dissection	20 (0.9)	72 (0.5)	0.046	9 (1.6)	18 (0.4)	< 0.001
No reflow	25 (1.1)	160 (1.2)	0.720	6 (1.0)	11 (0.2)	0.002
Major side branch occlusion	10 (0.4)	42 (0.3)	0.330	2 (0.3)	10 (0.2)	0.560
Heart failure	260 (11.3)	2014 (14.7)	< 0.001	24 (4.2)	142 (3.1)	0.200
CVA/stroke	5 (0.2)	61 (0.4)	0.110	1 (0.2)	5 (0.1)	0.680
New requirement for dialysis	17 (0.7)	77 (0.6)	0.310	1 (0.2)	0 (0.0)	0.005
Infection	20 (0.9)	65 (0.5)	0.017	3 (0.5)	4 (0.1)	0.008
Bleeding event in 72 h	126 (5.5)	684 (5.0)	0.360	28 (4.8)	166 (3.7)	0.160
In-hospital mortality	53 (2.3)	442 (3.2)	0.016	2 (0.4)	7 (0.2)	0.300
1-year mortality	235 (10.2)	1638 (12.0)	0.013	21 (3.6)	159 (3.5)	0.886

Values are expressed as *n* (%) or mean ± SD.

Abbreviations*:* C + , met criteria; C−, unmet criteria; CVA, cerebrovascular accident; IVUS, intravascular ultrasound; I + , IVUS-guided PCI, I−, angio-guided PCI; PCI, percutaneous coronary intervention.

The details of the univariate and multivariate analyses for each outcome model are presented in Supplementary Tables S1–S8. After adjusting for covariables, IVUS-guided PCI was identified as an independent predictor of the procedural success rate regardless of whether the criteria were met (OR 4.7, 95% CI 3.4–6.6, *P* < 0.001) or were not met (OR 2.7, 95% CI 1.3–5.7, *P* = 0.009). In addition, the IVUS-guided PCI was associated with a higher procedural complication rate in the C − group (adjusted OR 2.1, 95% CI 1.4–3.0, *P* < 0.001), but not in the C + group (adjusted OR 1.0, 95% CI 0.8–1.3, *P* = 0.74). IVUS-guided PCI was not related to the in-hospital mortality rate or 1-year mortality rate in either the C + or C − group.

### The propensity score-adjusted

A propensity model was constructed to balance confounders between IVUS-guided PCI (I +) and angio-guided PCI (I−). The absolute standardized mean differences ranged from 0.058 to 0.594 and 0.260 to 0.637 in C + and C− groups, see Table S9. After weighting by propensity score, the absolute standardized weight mean differences of these corresponding groups ranged from 0.005 to 0.040 and 0.009 to 0.128 indicating well balance of these confounders between IVUS-guided PCI groups. In addition, density plots also indicated that distributions of each covariate were well balanced between I + and I− groups, see Fig. S1a,b. Furthermore, a positivity assumption was checked by overlapping plots, indicating the probabilities of I + and I− were very much overlapped, see Fig. S2a,b.

**Figure 3:**
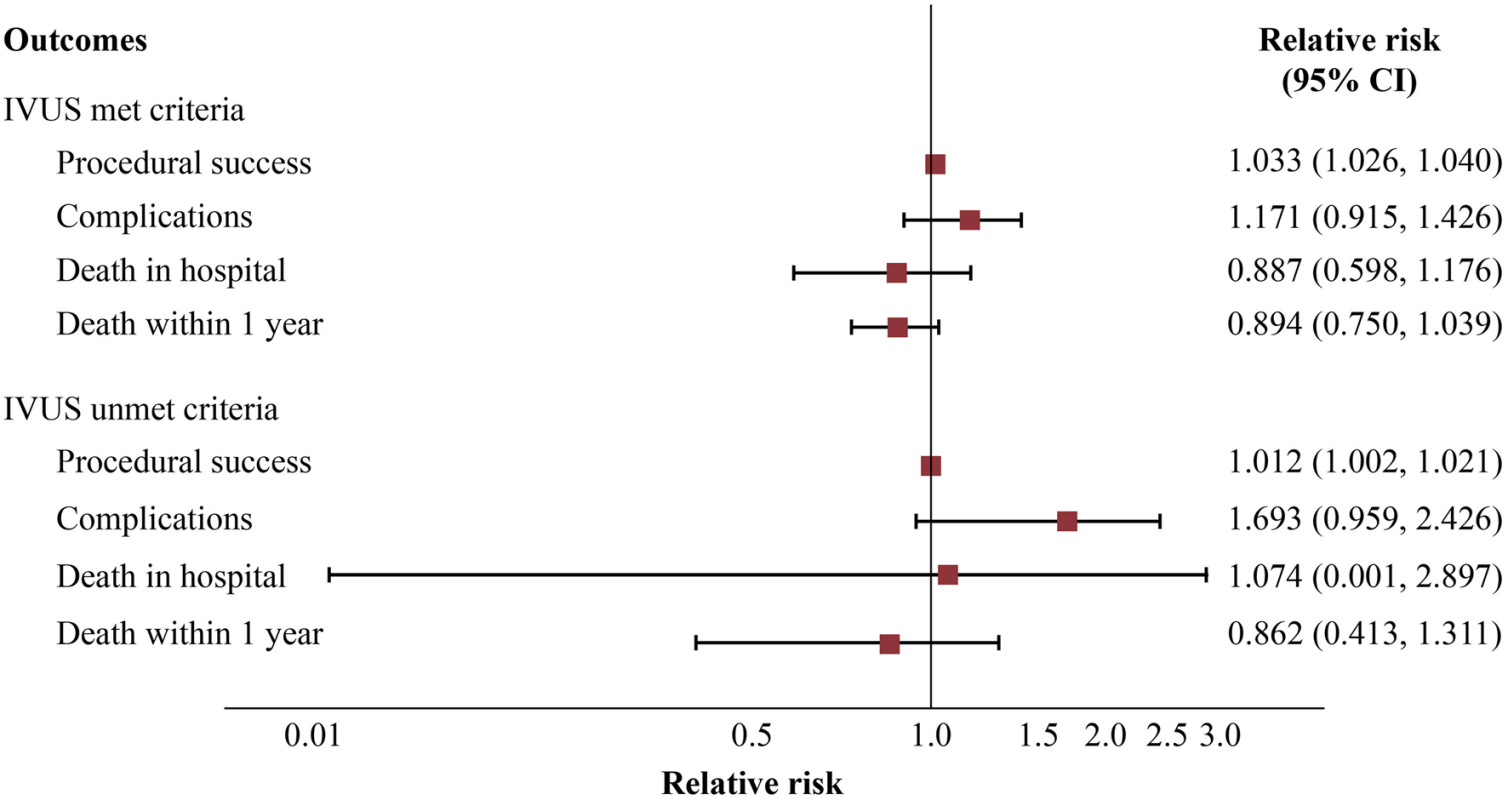
Relative risks of the primary and secondary endpoints by IVUS criteria groups: A propensity score analysis. Abbreviations: CI, confidence interval; IVUS, intravascular ultrasound.

**Figure 4:**
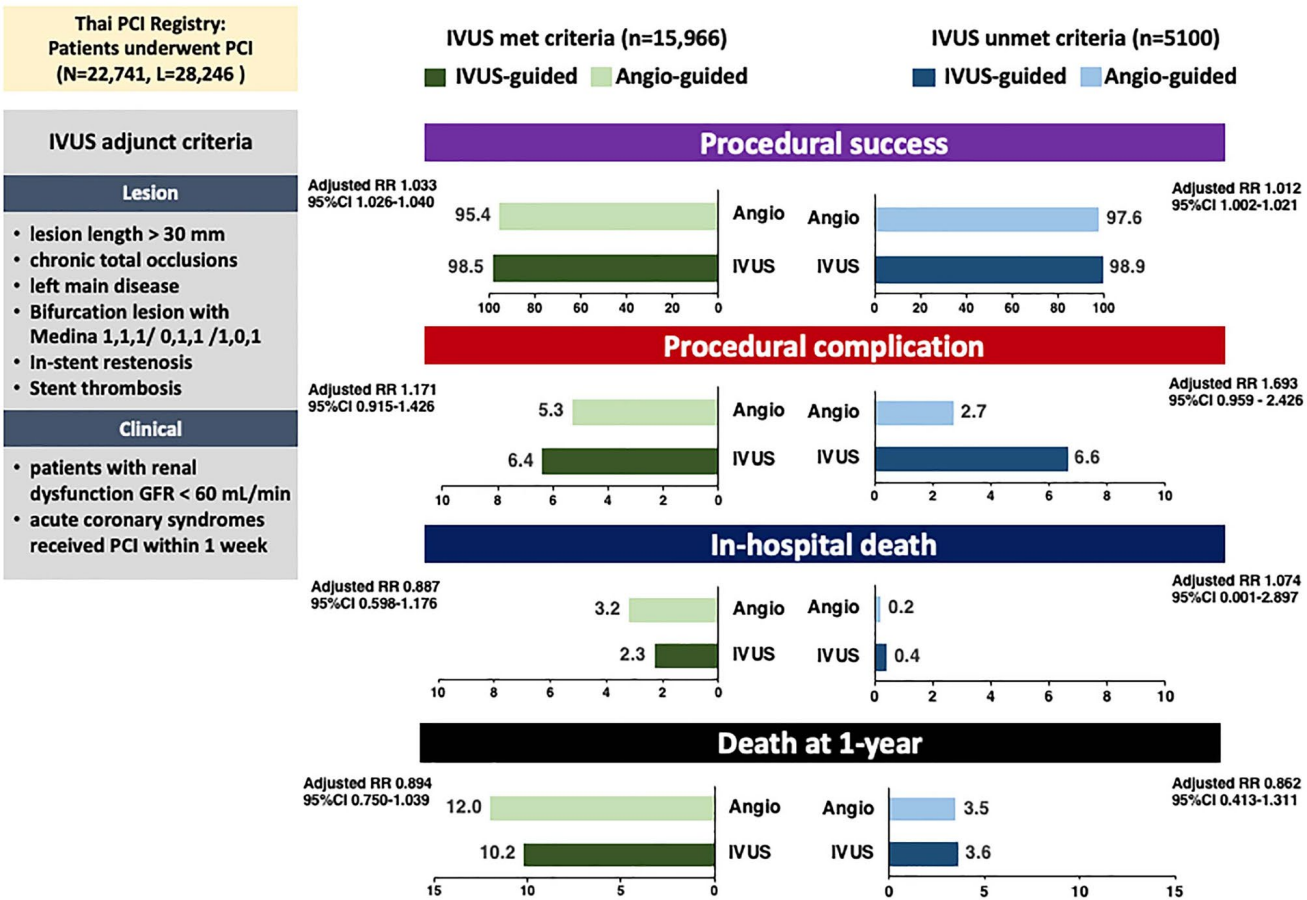
Graphic abstract. Abbreviation: CI, confidence interval; GFR, glomerular filtration rate; IVUS, intravascular ultrasound; PCI, percutaneous coronary intervention; RR, relative risk.

Risks, risk difference (RD), and relative risk (RR) along with 95% confidence intervals (CIs) of the primary and secondary endpoints were estimated based on propensity models in the C + and C − groups (Table 4 and Fig. 3). After adjusting for covariables by propensity model, IVUS-guided PCI was identified as an independent predictor of the procedural success rate regardless of whether the criteria were met and were not met with RRs (95% CI) of 1.033 (1.026, 1.040) and 1.012 (1.002, 1.021), respectively. IVUS-guided PCI was associated with procedural complication in both the C + /C− groups but none of them was significant with the RRs (95% CI) of 1.171 (0.915, 1.426) and 1.693 (0.959, 2.426), respectively. In addition, IVUS-guided PCI was related to lowering risks of the in-hospital mortality rate and 1-year mortality rate the C + with the RRs (95% CI) of 0.887 (0.598, 1.176) and 0.894 (0.750, 1.039), but none was significant. Conversely, IVUS-guided PCI in group C− was associated with higher risk of in-hospital death with the RR of 1.074 (0.001, 2.897), although this was not significant. Effects of IVUS-guided PCI on primary and secondary endpoints in patients who met and unmet criteria are summarized in the Fig. 4.

**Table 4:** Estimations of relative effects of IVUS-guided uses stratify by meet/unmeet IVUS criteria: A propensity score model by inverse-probability-weighted with regression adjustment.

	Interventions	Risk (95% CI)	RD (95% CI)	RR (95% CI)
**C + group**				
Procedural success	*IVUS-guided PCI (I* +*)*	0.985 (0.980, 0.991)	0.031 (0.025, 0.038)	1.033 (1.026, 1.040)
	*Angio-guided PCI (I-)*	0.954 (0.950, 0.958)	0	1
Complications	*IVUS-guided PCI (I* +*)*	0.063 (0.050, 0.076)	0.009 (-0.004, 0.023)	1.171 (0.915, 1.426)
	*Angio-guided PCI (I-)*	0.054 (0.050, 0.058)	0	1
Death in hospital	*IVUS-guided PCI (I* +*)*	0.027 (0.019, 0.036)	-0.003 (-0.012, 0.005)	0.887 (0.598, 1.176)
	*Angio-guided PCI (I-)*	0.031 (0.028, 0.033)	0	1
Death within 1 year	*IVUS-guided PCI (I* +*)*	0.105 (0.088, 0.121)	-0.012 (-0.030, 0.005)	0.894 (0.750, 1.039)
	*Angio-guided PCI (I-)*	0.117 (0.112, 0.123)	0	1
**C- group**				
Procedural success	*IVUS-guided PCI (I* +*)*	0.988 (0.979, 0.996)	0.012 (0.002, 0.021)	1.012 (1.002, 1.021)
	*Angio-guided PCI (I-)*	0.976 (0.971, 0.981)	0	1
Complications	*IVUS-guided PCI (I* +*)*	0.049 (0.029, 0.068)	0.020 (0.00001, 0.040)	1.693 (0.959, 2.426)
	*Angio-guided PCI (I-)*	0.029 (0.024, 0.034)	0	1
Death in hospital	*IVUS-guided PCI (I* +*)*	0.0016 (-0.0008, 0.0040)	0.0001(-0.0026,0.0028)	1.074 (0.001, 2.897)
	*Angio-guided PCI (I-)*	0.0015 (0.0004, 0.0026)	0	1
Death within 1 year	*IVUS-guided PCI (I* +*)*	0.030 (0.015, 0.045)	-0.005 (-0.021, 0.011)	0.862 (0.413, 1.311)
	*Angio-guided PCI (I-)*	0.035 (0.029, 0.040)	0	1

Abbreviations: C + , met criteria; C−, unmet criteria; CI, confidence interval; IVUS, intravascular ultrasound; I + , IVUS-guided PCI, I-, angio-guided PCI; PCI, percutaneous coronary intervention; RD, risk difference; RR, relative risk.

## Discussion

The present study was performed to investigate the procedural success, complication, in-hospital mortality, and 1-year mortality rates when the adjunctive use criteria were applied for IVUS-guided PCI in real-world data. The main findings of the present study were as follows: despite no ceiling for IVUS catheter reimbursement in Thailand, the penetrance of IVUS catheters was low, as only 14.5% of patients who fulfilled the AUC were treated with IVUS-guided PCI. After propensity score adjustment, procedural success was achieved at a significantly high rate for IVUS-guided PCI regardless of whether the criteria were met. IVUS-guided PCI trended to increase procedural complication rates in patients who did and did not meet the AUC but both were not significant. IVUS-guided PCI seemed to lower the risk of short- or medium-term mortality rates if the criteria were met, but this did not reach statistical significance.

The present study demonstrated that the IVUS-guided PCI was around 10% of the overall population. The adoption rates of IVUS-guided PCI in the current study were slightly higher than those in previous reports from the United Kingdom and USA, where IVUS was used in 4.3%^29^ and 5.6%^12^ of cases, respectively. On the one hand, significant variation in IVUS use was noted according to the data from Japan, where IVUS was used in 84.8% of cases^7^. According to the practice survey by the EAPCI and the Japanese Association of Cardiovascular Interventions and Therapeutics in 2018, a high cost and prolonged procedure were the common reasons for limiting the use of intracoronary imaging in clinical practice^30^. The present study showed that IVUS-guided PCI had extended fluoroscopic time of around 10.9 and 12.3 min in patients who met and unmet the adjunct use criteria, respectively. Another possible reason for the low rates of IVUS-guided PCI in Thailand may be explained by the operators' familiarity with IVUS image interpretation. About 52% of the interventional cardiology fellows in the United States reported no or rudimentary education in IVUS^31^. In contrast, data from UK showed that the operators who graduated after year 2000 were almost 15-fold higher than the operator graduated before 1990 for using IVUS-guided PCI^29^. The findings reflected that adequate IVUS training would lead to an improvement in adoption of IVUS-guided PCI. Unfortunately, the Thailand PCI registry did not collect the data regarding operators’ experience, therefore, we could not demonstrate the association between the operator generation and the use of IVUS- guided PCI.

In view of procedural complication, the current study showed that IVUS-guided PCI increased the procedural complication risks in both groups but were not significant from propensity analysis. Previously, data from Japan showed that IVUS has reduced the risk of flow impairing severe coronary dissection^7^ confined to elective procedures, but not among urgent/emergent PCIs. In contrast, the higher rate of coronary dissection in the Thai PCI registry was noted when compared with the data from the Japanese registry. It might be explained by the study protocol of Thai PCI registry that documented all visible dissection flaps from coronary angiography whereas the Japanese registry documented only flow-impairing dissection. In addition, there are also differences in operator skill in IVUS imaging between Japan and Thailand. In Japan, IVUS-guided PCI was used in over 90% of elective PCI procedures and in over 80% of the overall procedures^7^, whereas the rate of IVUS use in Thailand is approximately 10%. Therefore, the rate of coronary dissection could be higher due to the lack of familiarity of the operators with the IVUS image sizing algorithm. In the OPUS-CLASS (OCT Compared With IVUS in a Coronary Lesion Assessment) study^32^, measurement of the lumen diameter and lumen area of a phantom model were compared between IVUS and OCT. The lumen area measured by OCT was equivalent to the actual lumen area of the phantom model, whereas the measurement by IVUS was significantly greater, by 8%, than the value measured by OCT^32^. If the operators were not aware of this overestimation, coronary dissection could occur from stent or balloon oversizing leading to intramural haematoma in less diseased reference segments^33^. Previously, a nationwide inpatient database in the USA reported that IVUS-guided PCI was used in only 10.4% of PCI procedures for calcified coronary lesions^34^, and the IVUS-assisted procedure increased the overall cardiac complication rate (OR 1.25, CI 1.03–1.53, *P* = 0.025). Similar findings were also reported in the nationwide inpatient database in the USA for PCI in ST-segment elevation MI (OR 4.26, 95% CI 2.34–7.7, *P* < 0.01)^35^. The difference in IVUS usage may explain the different complication rates between high-volume (Japan) and low-volume (Thailand and USA) countries.

In the present study, IVUS-guided PCI did not improve short- or medium-term mortality rates after adjusting for covariables by propensity model and irrespective of whether the criteria were met. Our results contrasted with the pooled analysis of IVUS-XPL and ULTIMATE trials^6^, which showed a reduction of cardiac death in patients who were treated with IVUS-guided PCI compared with angio-guided PCI. However, it should be noted that the pooled analysis was an ad-hoc analysis in 2577 patients, including only patients with long lesions whereas the current study stratified the patients according to the AUC. In addition, pooled analysis reported cardiac mortality at 3-year follow-up^6^ while in contrast, the present analysis had clinical follow-up for 1 year. Regarding the real-world data of IVUS-guided PCI on the mortality, our study results were in line with previous reports that there was no improvement of mortality^29,36^ and MACE^29^ rates with IVUS-guided PCI.

Patients who did not meet the adjunct use criteria(C-) in the present study could be assumed as the representative for the case with non-complex lesions. The current analysis showed that IVUS-guided PCI in patients who did not meet the criteria (C− /I + group) had numerically higher procedural complication rate than that of patients who did not meet the criteria and underwent angio-guided PCI (C−/I−). A possible explanation for this might be the ad-hoc use of IVUS catheters after unfavourable angiographic findings, such as no-reflow phenomenon, coronary perforation, and coronary dissection, as demonstrated in Table 3.

## Limitations

The present study had several limitations. First, the Thai PCI registry was an observational study. The criteria were applied retrospectively to the study population. The decision to use IVUS-guided PCI was made at the operator’s discretion. Second, IVUS was not available in all participating centres. Thus, there would have been variations in IVUS skill among centres. In addition, centres with IVUS facilities tend to perform more complex procedures than those without such facilities. It is unclear whether IVUS use was a marker of more complex procedures or supported/altered the operator strategy, as the study protocol did not document the pre-procedural SYNTAX score. Third, a detailed analysis of the IVUS findings, such as plaque characteristics, minimal stent area, and stent expansion, was not included in the study protocol. Finally, other long-term outcomes, such as recurrent MI and urgent revascularization, were not available in the present study.

## Conclusion

The penetrance of IVUS-guided PCI was low despite clinical indications for IVUS usage in real-world data. Significant procedural success was achieved with IVUS-guided PCI regardless of whether the AUC were met. The procedural complication did not escalate when IVUS-guided PCI. IVUS-guided PCI seemed to reduce the mortality rates if the AUC were met, but this did not reach statistical significance.

## Supplementary Information

Supplementary Information.

## Data Availability

The data supporting the findings of this study are available from the corresponding author upon reasonable request.

## Abbreviations

ACS: Acute coronary syndrome
AUC: Adjunctive use criteria
CTO: Chronic total occlusion
IVUS: Intravascular ultrasound
OCT: Optical coherence tomography
PCI: Percutaneous coronary intervention
MI: Myocardial infarction
