# Author Correction: Impact of the adjunctive use criteria for intravascular ultrasound-guided percutaneous coronary intervention and clinical outcomes

**DOI:** 10.1038/s41598-023-29201-y

**Published:** 2023-02-02

**Authors:** Pannipa Suwannasom, Ply Chichareon, Worawut Roongsangmanoon, Artit Thongtanomkul, Anuchit Wongpen, Muenpetch Muenkaew, Anek Kanoksilp, Mann Chandavimol, Srun Kuanprasert, Ammarin Thakkinstian, Suphot Srimahachota, Nakarin Sansanayudh

**Affiliations:** 1 Division of Cardiology, Department of Internal Medicine, Faculty of Medicine, Chiang Mai University, Chiang Mai, Thailand; 2 Faculty of Medicine, Songklanakarind Hospital, Prince of Songkla University, Songkla, Thailand; 3 Faculty of Medicine, HRH Princess MahaChakri Sirindhorn Medical Center, Srinakharinwirot University, Nakhon Nayok, Thailand; 4 Maharaj Nakorn Si Thammarat Hospital, Nakorn Si Thammarat, Thailand; 5 Udonthani Hospital, Udonthani, Thailand; 6 Faculty of Medicine, Thammasat University Hospital, Thammasat University, Bangkok, Thailand; 7 Central Chest Institute of Thailand, Nonthaburi, Thailand; 8 Division of Cardiology, Department of Medicine, Faculty of Medicine Ramathibodi Hospital, Mahidol University, Bangkok, Thailand; 9 Department of Clinical Epidemiology and Biostatistics, Faculty of Medicine, Ramathibodi Hospital, Mahidol University, Bangkok, Thailand; 10 Division of Cardiovascular Diseases, Department of Medicine, King Chulalongkorn Memorial Hospital, Bangkok, Thailand; 11 Phramongkutklao College of Medicine, 315 Ratchawithi Rd, Khwaeng Thung Phaya Thai, Bangkok, 10400 Thailand

Correction to: *Scientific Reports* 10.1038/s41598-022-27250-3, published online 13 January 2023

The original version of this Article contained an error in the spelling of the author Ammarin Thakkinstian which was incorrectly given as Ammarin Thakkinstain.

The original Article has been corrected.

