# Supplementary material for: Impact of the adjunctive use criteria for intravascular ultrasound-guided percutaneous coronary intervention and clinical outcomes
Source: Sci Rep. 2023 Jan 13;13:711. doi: 10.1038/s41598-022-27250-3 (PMC9839682; doi:10.1038/s41598-022-27250-3)

**Supplementary table legends**

**Table S1** A univariate and multivariate logistics regression of factors associated with procedural success in patients who met IVUS criteria

**Table S2** A univariate and multivariate logistics regression of factors associated with complications in patients who met IVUS criteria

**Table S3** A univariate and multivariate logistics regression of factors associated with in hospital death in patients who met IVUS criteria

**Table S4** A univariate and multivariate logistics regression of factors associated with death in 1 year in patients who met IVUS criteria

**Table S5** A univariate and multivariate logistics regression of factors associated with procedural success in patients who did not meet IVUS criteria

**Table S6** A univariate and multivariate logistics regression of factors associated with complications in patients who did not meet IVUS criteria

**Table S7** A univariate and multivariate logistics regression of factors associated with in hospital death in patients who did not meet IVUS criteria

**Table S8** A univariate and multivariate logistics regression of factors associated with death in 1 year in patients who did not meet IVUS criteria

**Table S9** Balance of factors associated with IVUS-guided allocations

**Figure S1.** Balance plot of kernel density of each covariate by IVUS vs non-IVUS groups stratify by IVUS criteria groups.

**Figure S2.** Overlapping plot: Estimating densities of the probability of IVUS and non-IVUS uses to assess if each individual patient has a positive probability of applying and not applying IVUS.

Table S1. Factors associated with procedural success in patients who met IVUS criteria

| Characteristics | Procedural success | | Univariate | | Multivariate | |
| --- | --- | --- | --- | --- | --- | --- |
|  | success | fail | OR (95% CI) | P value | OR (95% CI) | P value |
|  | n = 15,327 | n = 639 |  |  |  |  |
| Indication-IVUS, n (%) |  |  |  |  |  |  |
| C-matched-IVUS | 2,263 (98.1) | 44 (1.9) | 2.3 (1.7, 3.2) | <0.001 | 4.7 (3.4, 6.6) | <0.001 |
| C-matched-nonIVUS | 13,064 (95.6) | 595 (4.4) | 1 |  | 1 |  |
| Chronic total occlusion, n (%) |  |  |  |  |  |  |
| Yes | 1,489 (80.1) | 370 (19.9) | 0.08 (0.07, 0.09) | <0.001 | 0.07 (0.06, 0.09) | <0.001 |
| No | 13,838 (98.1) | 269 (1.9) | 1 |  | 1 |  |
| Total volume of contrast, ml, median (range) | 100.0 (10.0, 600.0) | 120.0 (20.0, 480.0) | 0.994 (0.992, 0.995) | <0.001 | 0.998 (0.996, 0.999) | 0.001 |
| Lesion complexity, n (%) |  |  |  |  |  |  |
| B2 or C | 12,401 (95.4) | 597 (4.6) | 0.3 (0.2, 0.4) | <0.001 | 0.4 (0.3, 0.6) | <0.001 |
| A or B1 | 2,819 (98.8) | 35 (1.2) | 1 |  | 1 |  |
| CAD presentation, n (%) |  |  |  |  |  |  |
| STEMI | 5,568 (97.9) | 121 (2.1) | 2.6 (2.1, 3.3) | <0.001 | 0.9 (0.7, 1.1) | 0.256 |
| NSTEMI / Unstable Angina | 4,716 (95.4) | 228 (4.6) | 1.2 (1.0, 1.4) | 0.056 | 0.7 (0.6, 0.9) | 0.003 |
| Stable CAD | 5,043 (94.6) | 290 (5.4) | 1 |  | 1 |  |
| Prior MI, n (%) |  |  |  |  |  |  |
| Yes | 3,071 (93.9) | 199 (6.1) | 0.6 (0.5, 0.7) | <0.001 | 0.7 (0.6, 0.8) | <0.001 |
| No | 12,256 (96.5) | 440 (3.5) | 1 |  | 1 |  |
| Prior CABG, n (%) |  |  |  |  |  |  |
| Yes | 229 (91.6) | 21 (8.4) | 0.4 (0.3, 0.7) | <0.001 | 0.5 (0.3, 0.9) | 0.010 |
| No | 15,098 (96.1) | 618 (3.9) | 1 |  | 1 |  |
| CKD, n (%) |  |  |  |  |  |  |
| Yes | 6,459 (95.5) | 302 (4.5) | 0.8 (0.7, 1.0) | 0.010 | 0.6 (0.5, 0.7) | <0.001 |
| No | 8,868 (96.3) | 337 (3.7) | 1 |  | 1 |  |
| Gender, n (%) |  |  |  |  |  |  |
| Female | 4,820 (96.1) | 194 (3.9) | 1.1 (0.9, 1.2) | 0.562 |  |  |
| Male | 10,507 (95.9) | 445 (4.1) | 1 |  |  |  |
| Age, years, mean (SD) | 64.7 (11.9) | 65.3 (12.0) | 0.996 (0.989, 1.002) | 0.210 |  |  |
| Diabetes mellitus, n (%) |  |  |  |  |  |  |
| Yes | 7,037 (95.8) | 305 (4.2) | 0.9 (0.8, 1.1) | 0.366 |  |  |
| No | 8,290 (96.1) | 334 (3.9) | 1 |  |  |  |
| On dialysis, n (%) |  |  |  |  |  |  |
| Yes | 720 (96.0) | 30 (4.0) | 1.0 (0.7, 1.5) | 0.997 |  |  |
| No | 14,607 (96.0) | 609 (4.0) | 1 |  |  |  |
| Hypertension, n (%) |  |  |  |  |  |  |
| Yes | 10,080 (95.5) | 472 (4.5) | 0.7 (0.6, 0.8) | <0.001 |  |  |
| No | 5,247 (96.9) | 167 (3.1) | 1 |  |  |  |
| Dyslipidemia, n (%) |  |  |  |  |  |  |
| Yes | 9,580 (95.6) | 440 (4.4) | 0.8 (0.6, 0.9) | 0.001 |  |  |
| No | 5,747 (96.7) | 199 (3.3) | 1 |  |  |  |
| Peripheral Arterial Disease, n (%) |  |  |  |  |  |  |
| Yes | 238 (93.7) | 16 (6.3) | 0.6 (0.4, 1.0) | 0.062 |  |  |
| No | 15,089 (96.0) | 623 (4.0) | 1 |  |  |  |
| Prior PCI, n (%) |  |  |  |  |  |  |
| Yes | 3,659 (94.7) | 205 (5.3) | 0.7 (0.6, 0.8) | <0.001 |  |  |
| No | 11,668 (96.4) | 434 (3.6) | 1 |  |  |  |
| Cardiogenic shock at start of PCI, n (%) |  |  |  |  |  |  |
| Yes | 1,485 (95.6) | 69 (4.4) | 0.9 (0.7, 1.1) | 0.354 |  |  |
| No | 13,842 (96.0) | 570 (4.0) | 1 |  |  |  |
| LVEF, %, mean (SD) | 50.5 (15.3) | 47.1 (16.3) | 1.014 (1.008, 1.020) | <0.001 |  |  |
| Bifurcation lesion, n (%) |  |  |  |  |  |  |
| Yes | 2,136 (96.9) | 68 (3.1) | 1.4 (1.1, 1.8) | 0.019 |  |  |
| No | 13,086 (95.9) | 566 (4.1) | 1 |  |  |  |
| Previously treated lesion, n (%) |  |  |  |  |  |  |
| Yes | 757 (93.6) | 52 (6.4) | 0.6 (0.4, 0.8) | <0.001 |  |  |
| No | 14,542 (96.1) | 587 (3.9) | 1 |  |  |  |
| Initial access site, n (%) |  |  |  |  |  |  |
| Brachial only and other | 34 (97.1) | 1 (2.9) | 1.1 (0.1, 7.9) | 0.945 |  |  |
| Femoral only | 8,223 (95.4) | 393 (4.6) | 0.7 (0.6, 0.8) | <0.001 |  |  |
| Combination | 316 (90.8) | 32 (9.2) | 0.3 (0.2, 0.5) | <0.001 |  |  |
| Radial only | 6,754 (96.9) | 213 (3.1) | 1 |  |  |  |
| Plaque modification, n (%) |  |  |  |  |  |  |
| Yes | 734 (96.8) | 24 (3.2) | 1.3 (0.9, 2.0) | 0.230 |  |  |
| No | 14,547 (96.0) | 613 (4.0) | 1 |  |  |  |
| Fluoroscopy time, minutes, median (range) | 12.5 (0.1, 770.0) | 29.0 (1.4, 200.0) | 0.971 (0.968, 0.974) | <0.001 |  |  |

Table S2. Factors associated with complications in patients who met IVUS criteria

| Characteristics | Complications | | Univariate | | Multivariate | |
| --- | --- | --- | --- | --- | --- | --- |
|  | Yes | No | OR (95% CI) | P value | OR (95% CI) | P value |
|  | n = 871 | n = 15,095 |  |  |  |  |
| Indication-IVUS, n (%) |  |  |  |  |  |  |
| C-matched-IVUS | 147 (6.4) | 2,160 (93.6) | 1.2 (1.0, 1.5) | 0.036 | 1.0 (0.8, 1.3) | 0.738 |
| C-matched-nonIVUS | 724 (5.3) | 12,935 (94.7) | 1 |  | 1 |  |
| Cardiogenic shock at start of PCI, n (%) |  |  |  |  |  |  |
| Yes | 192 (12.4) | 1,362 (87.6) | 2.9 (2.4, 3.4) | <0.001 | 2.2 (1.8, 2.6) | <0.001 |
| No | 679 (4.7) | 13,733 (95.3) | 1 |  | 1 |  |
| CAD presentation, n (%) |  |  |  |  |  |  |
| STEMI | 457 (8.0) | 5,232 (92.0) | 2.0 (1.7, 2.3) | <0.001 | 2.2 (1.8, 2.7) | <0.001 |
| NSTEMI / Unstable Angina | 187 (3.8) | 4,757 (96.2) | 0.9 (0.7, 1.1) | 0.222 | 1.0 (0.8, 1.2) | 0.787 |
| Stable CAD | 227 (4.3) | 5,106 (95.7) | 1 |  | 1 |  |
| Age, years, mean (SD) | 65.6 (12.5) | 64.6 (11.9) | 1.007 (1.001, 1.013) | 0.024 | 1.011 (1.005, 1.017) | <0.001 |
| Lesion complexity, n (%) |  |  |  |  |  |  |
| B2 or C | 786 (6.0) | 12,212 (94.0) | 2.3 (1.8, 2.9) | <0.001 | 2.0 (1.6, 2.6) | <0.001 |
| A or B1 | 79 (2.8) | 2,775 (97.2) | 1 |  | 1 |  |
| Fluoroscopy time, minutes, median (range) | 17.1 (1.5, 182.3) | 12.6 (0.1, 770.0) | 1.011 (1.008, 1.014) | <0.001 | 1.007 (1.003, 1.010) | <0.001 |
| Total volume of contrast, ml, median (range) | 110.0 (20.0, 582.0) | 100.0 (10.0, 600.0) | 1.005 (1.003, 1.006) | <0.001 | 1.004 (1.003, 1.005) | <0.001 |
| Bifurcation lesion, n (%) |  |  |  |  |  |  |
| Yes | 171 (7.8) | 2,033 (92.2) | 1.6 (1.3, 1.9) | <0.001 | 1.5 (1.2, 1.8) | <0.001 |
| No | 697 (5.1) | 12,955 (94.9) | 1 |  | 1 |  |
| Hypertension, n (%) |  |  |  |  |  |  |
| Yes | 513 (4.9) | 10,039 (95.1) | 0.7 (0.6, 0.8) | <0.001 | 0.8 (0.7, 0.9) | 0.007 |
| No | 358 (6.6) | 5,056 (93.4) | 1 |  | 1 |  |
| Chronic total occlusion, n (%) |  |  |  |  |  |  |
| Yes | 125 (6.7) | 1,734 (93.3) | 1.3 (1.1, 1.6) | 0.011 | 1.3 (1.0, 1.6) | 0.028 |
| No | 746 (5.3) | 13,361 (94.7) | 1 |  | 1 |  |
| Gender, n (%) |  |  |  |  |  |  |
| Female | 284 (5.7) | 4,730 (94.3) | 1.1 (0.9, 1.2) | 0.432 |  |  |
| Male | 587 (5.4) | 10,365 (94.6) | 1 |  |  |  |
| Diabetes mellitus, n (%) |  |  |  |  |  |  |
| Yes | 400 (5.4) | 6,942 (94.6) | 1.0 (0.9, 1.1) | 0.970 |  |  |
| No | 471 (5.5) | 8,153 (94.5) | 1 |  |  |  |
| CKD, n (%) |  |  |  |  |  |  |
| Yes | 352 (5.2) | 6,409 (94.8) | 0.9 (0.8, 1.1) | 0.235 |  |  |
| No | 519 (5.6) | 8,686 (94.4) | 1 |  |  |  |
| On dialysis, n (%) |  |  |  |  |  |  |
| Yes | 21 (2.8) | 729 (97.2) | 0.5 (0.3, 0.8) | 0.001 |  |  |
| No | 850 (5.6) | 14,366 (94.4) | 1 |  |  |  |
| Dyslipidemia, n (%) |  |  |  |  |  |  |
| Yes | 510 (5.1) | 9,510 (94.9) | 0.8 (0.7, 1.0) | 0.008 |  |  |
| No | 361 (6.1) | 5,585 (93.9) | 1 |  |  |  |
| Peripheral Arterial Disease, n (%) |  |  |  |  |  |  |
| Yes | 15 (5.9) | 239 (94.1) | 1.1 (0.6, 1.8) | 0.750 |  |  |
| No | 856 (5.4) | 14,856 (94.6) | 1 |  |  |  |
| Prior PCI, n (%) |  |  |  |  |  |  |
| Yes | 166 (4.3) | 3,698 (95.7) | 0.7 (0.6, 0.9) | <0.001 |  |  |
| No | 705 (5.8) | 11,397 (94.2) | 1 |  |  |  |
| Prior CABG, n (%) |  |  |  |  |  |  |
| Yes | 11 (4.4) | 239 (95.6) | 0.8 (0.4, 1.5) | 0.460 |  |  |
| No | 860 (5.5) | 14,856 (94.5) | 1 |  |  |  |
| Prior MI, n (%) |  |  |  |  |  |  |
| Yes | 196 (6.0) | 3,074 (94.0) | 1.1 (1.0, 1.3) | 0.129 |  |  |
| No | 675 (5.3) | 12,021 (94.7) | 1 |  |  |  |
| LVEF, %, mean (SD) | 47.9 (14.9) | 50.5 (15.4) | 0.989 (0.984, 0.995) | <0.001 |  |  |
| Previously treated lesion, n (%) |  |  |  |  |  |  |
| Yes | 48 (5.9) | 761 (94.1) | 1.1 (0.8, 1.5) | 0.548 |  |  |
| No | 823 (5.4) | 14,306 (94.6) | 1 |  |  |  |
| Initial access site, n (%) |  |  |  |  |  |  |
| Brachial only and other | 2 (5.7) | 33 (94.3) | 1.1 (0.3, 4.7) | 0.878 |  |  |
| Femoral only | 478 (5.5) | 8,138 (94.5) | 1.1 (0.9, 1.2) | 0.260 |  |  |
| Combination | 33 (9.5) | 315 (90.5) | 1.9 (1.3, 2.8) | 0.001 |  |  |
| Radial only | 358 (5.1) | 6,609 (94.9) | 1 |  |  |  |
| Plaque modification, n (%) |  |  |  |  |  |  |
| Yes | 46 (6.1) | 712 (93.9) | 1.1 (0.8, 1.5) | 0.440 |  |  |
| No | 821 (5.4) | 14,339 (94.6) | 1 |  |  |  |

Table S3. Factors associated with in hospital death in patients who met IVUS criteria

| Characteristics | Death | | Univariate | | Multivariate | |
| --- | --- | --- | --- | --- | --- | --- |
|  | Yes | No | OR (95% CI) | P value | OR (95% CI) | P value |
|  | n = 495 | n = 15,471 |  |  |  |  |
| Indication-IVUS, n (%) |  |  |  |  |  |  |
| C-matched-IVUS | 53 (2.3) | 2,254 (97.7) | 0.7 (0.5, 0.9) | 0.017 | 1.1 (0.8, 1.5) | 0.536 |
| C-matched-nonIVUS | 442 (3.2) | 13,217 (96.8) | 1 |  | 1 |  |
| Cardiogenic shock at start of PCI, n (%) |  |  |  |  |  |  |
| Yes | 303 (19.5) | 1,251 (80.5) | 17.9 (14.8, 21.7) | <0.001 | 10.8 (8.8, 13.3) | <0.001 |
| No | 192 (1.3) | 14,220 (98.7) | 1 |  | 1 |  |
| CAD presentation, n (%) |  |  |  |  |  |  |
| ACS | 469 (4.4) | 10,164 (95.6) | 9.4 (6.3, 14.0) | <0.001 | 5.7 (3.8, 8.6) | <0.001 |
| Stable CAD | 26 (0.5) | 5,307 (99.5) | 1 |  | 1 |  |
| CKD, n (%) |  |  |  |  |  |  |
| Yes | 346 (5.1) | 6,415 (94.9) | 3.3 (2.7, 4.0) | <0.001 | 2.4 (1.9, 3.0) | <0.001 |
| No | 149 (1.6) | 9,056 (98.4) | 1 |  | 1 |  |
| Age, years, mean (SD) | 70.5 (12.6) | 64.5 (11.8) | 1.045 (1.037, 1.054) | <0.001 | 1.03 (1.02, 1.04) | <0.001 |
| Femoral access, n (%) |  |  |  |  |  |  |
| Yes | 389 (4.4) | 8,528 (95.6) | 3.0 (2.4, 3.7) | <0.001 | 2.1 (1.7, 2.7) | <0.001 |
| No | 106 (1.5) | 6,943 (98.5) | 1 |  | 1 |  |
| Diabetes mellitus, n (%) |  |  |  |  |  |  |
| Yes | 287 (3.9) | 7,055 (96.1) | 1.6 (1.4, 2.0) | <0.001 | 1.4 (1.1, 1.7) | 0.001 |
| No | 208 (2.4) | 8,416 (97.6) | 1 |  | 1 |  |
| Lesion complexity, n (%) |  |  |  |  |  |  |
| B2 or C | 422 (3.2) | 12,576 (96.8) | 1.3 (1.0, 1.7) | 0.027 | 1.4 (1.1, 1.9) | 0.010 |
| A or B1 | 70 (2.5) | 2,784 (97.5) | 1 |  | 1 |  |
| On dialysis, n (%) |  |  |  |  |  |  |
| Yes | 34 (4.5) | 716 (95.5) | 1.5 (1.1, 2.2) | 0.021 | 1.5 (1.0, 2.2) | 0.048 |
| No | 461 (3.0) | 14,755 (97.0) | 1 |  | 1 |  |
| Dyslipidemia, n (%) |  |  |  |  |  |  |
| Yes | 243 (2.4) | 9,777 (97.6) | 0.6 (0.5, 0.7) | <0.001 | 0.7 (0.6, 0.9) | 0.004 |
| No | 252 (4.2) | 5,694 (95.8) | 1 |  | 1 |  |
| Gender, n (%) |  |  |  |  |  |  |
| Female | 196 (3.9) | 4,818 (96.1) | 1.4 (1.2, 1.7) | <0.001 |  |  |
| Male | 299 (2.7) | 10,653 (97.3) | 1 |  |  |  |
| Hypertension, n (%) |  |  |  |  |  |  |
| Yes | 304 (2.9) | 10,248 (97.1) | 0.8 (0.7, 1.0) | 0.026 |  |  |
| No | 191 (3.5) | 5,223 (96.5) | 1 |  |  |  |
| Peripheral Arterial Disease, n (%) |  |  |  |  |  |  |
| Yes | 12 (4.7) | 242 (95.3) | 1.6 (0.9, 2.8) | 0.135 |  |  |
| No | 483 (3.1) | 15,229 (96.9) | 1 |  |  |  |
| Prior PCI, n (%) |  |  |  |  |  |  |
| Yes | 51 (1.3) | 3,813 (98.7) | 0.4 (0.3, 0.5) | <0.001 |  |  |
| No | 444 (3.7) | 11,658 (96.3) | 1 |  |  |  |
| Prior CABG, n (%) |  |  |  |  |  |  |
| Yes | 9 (3.6) | 241 (96.4) | 1.2 (0.6, 2.3) | 0.646 |  |  |
| No | 486 (3.1) | 15,230 (96.9) | 1 |  |  |  |
| Prior MI, n (%) |  |  |  |  |  |  |
| Yes | 66 (2.0) | 3,204 (98.0) | 0.6 (0.5, 0.8) | <0.001 |  |  |
| No | 429 (3.4) | 12,267 (96.6) | 1 |  |  |  |
| LVEF, %, mean (SD) | 38.4 (15.6) | 50.6 (15.2) | 0.95 (0.94, 0.96) | <0.001 |  |  |
| Bifurcation lesion, n (%) |  |  |  |  |  |  |
| Yes | 77 (3.5) | 2,127 (96.5) | 1.2 (0.9, 1.5) | 0.247 |  |  |
| No | 414 (3.0) | 13,238 (97.0) | 1 |  |  |  |
| Previously treated lesion, n (%) |  |  |  |  |  |  |
| Yes | 19 (2.3) | 790 (97.7) | 0.7 (0.5, 1.2) | 0.208 |  |  |
| No | 475 (3.1) | 14,654 (96.9) | 1 |  |  |  |
| Chronic total occlusion, n (%) |  |  |  |  |  |  |
| Yes | 30 (1.6) | 1,829 (98.4) | 0.5 (0.3, 0.7) | <0.001 |  |  |
| No | 465 (3.3) | 13,642 (96.7) | 1 |  |  |  |
| Plaque modification, n (%) |  |  |  |  |  |  |
| Yes | 24 (3.2) | 734 (96.8) | 1.0 (0.7, 1.6) | 0.878 |  |  |
| No | 465 (3.1) | 14,695 (96.9) | 1 |  |  |  |
| Fluoroscopy time, minutes, median (range) | 14.0 (1.3, 182.3) | 13.0 (0.1, 770.0) | 1.001 (0.997, 1.006) | 0.522 |  |  |
| Total volume of contrast, ml, median (range) | 100.0 (20.0, 420.0) | 100.0 (10.0, 600.0) | 1.001 (0.999, 1.003) | 0.248 |  |  |

Table S4. Factors associated with death in 1 year in patients who met IVUS criteria

| Characteristics | Death in 1 year | | Univariate | | Multivariate | |
| --- | --- | --- | --- | --- | --- | --- |
|  | Yes | No | OR (95% CI) | P value | OR (95% CI) | P value |
|  | n = 1,873 | n = 14,093 |  |  |  |  |
| Indication-IVUS, n (%) |  |  |  |  |  |  |
| C-matched-IVUS | 235 (10.2) | 2,072 (89.8) | 0.8 (0.7, 1.0) | 0.013 | 0.9 (0.8, 1.1) | 0.263 |
| C-matched-nonIVUS | 1,638 (12.0) | 12,021 (88.0) | 1 |  | 1 |  |
| Cardiogenic shock at start of PCI, n (%) |  |  |  |  |  |  |
| Yes | 543 (34.9) | 1,011 (65.1) | 5.3 (4.7, 5.9) | <0.001 | 4.6 (4.0, 5.3) | <0.001 |
| No | 1,330 (9.2) | 13,082 (90.8) | 1 |  | 1 |  |
| CKD, n (%) |  |  |  |  |  |  |
| Yes | 1,282 (19.0) | 5,479 (81.0) | 3.4 (3.1, 3.8) | <0.001 | 2.2 (2.0, 2.5) | <0.001 |
| No | 591 (6.4) | 8,614 (93.6) | 1 |  | 1 |  |
| Age, years, mean (SD) | 70.3 (11.9) | 63.9 (11.7) | 1.050 (1.045, 1.054) | <0.001 | 1.038 (1.033, 1.043) | <0.001 |
| On dialysis, n (%) |  |  |  |  |  |  |
| Yes | 219 (29.2) | 531 (70.8) | 3.4 (2.9, 4.0) | <0.001 | 2.6 (2.2, 3.2) | <0.001 |
| No | 1,654 (10.9) | 13,562 (89.1) | 1 |  | 1 |  |
| CAD presentation, n (%) |  |  |  |  |  |  |
| ACS | 1,430 (13.4) | 9,203 (86.6) | 1.7 (1.5, 1.9) | <0.001 | 1.7 (1.5, 2.0) | <0.001 |
| Stable CAD | 443 (8.3) | 4,890 (91.7) | 1 |  | 1 |  |
| Femoral access, n (%) |  |  |  |  |  |  |
| Yes | 1,299 (14.6) | 7,618 (85.4) | 1.9 (1.7, 2.1) | <0.001 | 1.4 (1.3, 1.6) | <0.001 |
| No | 574 (8.1) | 6,475 (91.9) | 1 |  | 1 |  |
| Diabetes mellitus, n (%) |  |  |  |  |  |  |
| Yes | 1,067 (14.5) | 6,275 (85.5) | 1.6 (1.5, 1.8) | <0.001 | 1.4 (1.3, 1.6) | <0.001 |
| No | 806 (9.3) | 7,818 (90.7) | 1 |  | 1 |  |
| Dyslipidemia, n (%) |  |  |  |  |  |  |
| Yes | 1,065 (10.6) | 8,955 (89.4) | 0.76 (0.69, 0.83) | <0.001 | 0.7 (0.6, 0.8) | <0.001 |
| No | 808 (13.6) | 5,138 (86.4) | 1 |  | 1 |  |
| Fluoroscopy time, minutes, median (range) | 14.1 (1.1, 770.0) | 12.7 (0.1, 540.0) | 1.004 (1.002, 1.006) | <0.001 | 1.007 (1.004, 1.010) | <0.001 |
| Peripheral Arterial Disease, n (%) |  |  |  |  |  |  |
| Yes | 64 (25.2) | 190 (74.8) | 2.6 (1.9, 3.5) | <0.001 | 2.0 (1.4, 2.7) | <0.001 |
| No | 1,809 (11.5) | 13,903 (88.5) | 1 |  | 1 |  |
| Lesion complexity, n (%) |  |  |  |  |  |  |
| B2 or C | 1,576 (12.1) | 11,422 (87.9) | 1.3 (1.1, 1.4) | 0.001 | 1.3 (1.1, 1.5) | <0.001 |
| A or B1 | 281 (9.8) | 2,573 (90.2) | 1 |  | 1 |  |
| Gender, n (%) |  |  |  |  |  |  |
| Female | 741 (14.8) | 4,273 (85.2) | 1.5 (1.4, 1.7) | <0.001 | 1.1 (1.0, 1.3) | 0.018 |
| Male | 1,132 (10.3) | 9,820 (89.7) | 1 |  | 1 |  |
| Hypertension, n (%) |  |  |  |  |  |  |
| Yes | 1,331 (12.6) | 9,221 (87.4) | 1.3 (1.2, 1.4) | <0.001 |  |  |
| No | 542 (10.0) | 4,872 (90.0) | 1 |  |  |  |
| Prior PCI, n (%) |  |  |  |  |  |  |
| Yes | 368 (9.5) | 3,496 (90.5) | 0.74 (0.66, 0.84) | <0.001 |  |  |
| No | 1,505 (12.4) | 10,597 (87.6) | 1 |  |  |  |
| Prior CABG, n (%) |  |  |  |  |  |  |
| Yes | 31 (12.4) | 219 (87.6) | 1.1 (0.7, 1.6) | 0.741 |  |  |
| No | 1,842 (11.7) | 13,874 (88.3) | 1 |  |  |  |
| Prior MI, n (%) |  |  |  |  |  |  |
| Yes | 360 (11.0) | 2,910 (89.0) | 0.9 (0.8, 1.0) | 0.150 |  |  |
| No | 1,513 (11.9) | 11,183 (88.1) | 1 |  |  |  |
| LVEF, %, mean (SD) | 42.2 (15.9) | 51.3 (15.0) | 0.961 (0.957, 0.966) | <0.001 |  |  |
| Bifurcation lesion, n (%) |  |  |  |  |  |  |
| Yes | 272 (12.3) | 1,932 (87.7) | 1.1 (0.9, 1.2) | 0.327 |  |  |
| No | 1,586 (11.6) | 12,066 (88.4) | 1 |  |  |  |
| Previously treated lesion, n (%) |  |  |  |  |  |  |
| Yes | 101 (12.5) | 708 (87.5) | 1.1 (0.9, 1.3) | 0.499 |  |  |
| No | 1,770 (11.7) | 13,359 (88.3) | 1 |  |  |  |
| Chronic total occlusion, n (%) |  |  |  |  |  |  |
| Yes | 152 (8.2) | 1,707 (91.8) | 0.6 (0.5, 0.8) | <0.001 |  |  |
| No | 1,721 (12.2) | 12,386 (87.8) | 1 |  |  |  |
| Plaque modification, n (%) |  |  |  |  |  |  |
| Yes | 116 (15.3) | 642 (84.7) | 1.4 (1.1, 1.7) | 0.002 |  |  |
| No | 1,747 (11.5) | 13,413 (88.5) | 1 |  |  |  |
| Total volume of contrast, ml, median (range) | 100.0 (15.0, 420.0) | 100.0 (10.0, 600.0) | 0.9991 (0.9981, 1.0000) | 0.045 |  |  |

Table S5. Factors associated with procedural success in patients who did not meet IVUS criteria

| Characteristics | Procedural success | | Univariate | | Multivariate | |
| --- | --- | --- | --- | --- | --- | --- |
|  | success | fail | OR (95% CI) | P value | OR (95% CI) | P value |
|  | n = 4,984 | n = 116 |  |  |  |  |
| Indication-IVUS, n (%) |  |  |  |  |  |  |
| C-unmatched-IVUS | 570 (98.6) | 8 (1.4) | 1.7 (0.8, 3.6) | 0.132 | 2.7 (1.3, 5.7) | 0.009 |
| C-unmatched-nonIVUS | 4,414 (97.6) | 108 (2.4) | 1 |  | 1 |  |
| Lesion complexity, n (%) |  |  |  |  |  |  |
| B2 or C | 3,387 (97.1) | 102 (2.9) | 0.3 (0.2, 0.5) | <0.001 | 0.3 (0.2, 0.6) | <0.001 |
| A or B1 | 1,564 (99.2) | 13 (0.8) | 1 |  | 1 |  |
| Fluoroscopy time, minutes, median (range) | 11.2 (0.5, 910.0) | 19.6 (3.0, 143.2) | 0.98 (0.97, 0.99) | <0.001 | 0.984 (0.976, 0.993) | 0.001 |
| Total volume of contrast, ml, median (range) | 100.0 (20.0, 600.0) | 112.5 (30.0, 400.0) | 0.993 (0.991, 0.996) | <0.001 | 0.996 (0.993, 0.999) | 0.013 |
| Gender, n (%) |  |  |  |  |  |  |
| Female | 1,431 (97.8) | 32 (2.2) | 1.1 (0.7, 1.6) | 0.791 |  |  |
| Male | 3,553 (97.7) | 84 (2.3) | 1 |  |  |  |
| Age, years, mean (SD) | 62.3 (10.7) | 62.5 (10.0) | 1.00 (0.98, 1.02) | 0.862 |  |  |
| Diabetes mellitus, n (%) |  |  |  |  |  |  |
| Yes | 1,796 (97.7) | 42 (2.3) | 1.0 (0.7, 1.5) | 0.970 |  |  |
| No | 3,188 (97.7) | 74 (2.3) | 1 |  |  |  |
| Hypertension, n (%) |  |  |  |  |  |  |
| Yes | 3,483 (97.6) | 86 (2.4) | 0.8 (0.5, 1.2) | 0.324 |  |  |
| No | 1,501 (98.0) | 30 (2.0) | 1 |  |  |  |
| Dyslipidemia, n (%) |  |  |  |  |  |  |
| Yes | 3,645 (97.9) | 78 (2.1) | 1.3 (0.9, 2.0) | 0.159 |  |  |
| No | 1,339 (97.2) | 38 (2.8) | 1 |  |  |  |
| Peripheral Arterial Disease, n (%) |  |  |  |  |  |  |
| Yes | 75 (96.2) | 3 (3.8) | 0.6 (0.2, 1.9) | 0.354 |  |  |
| No | 4,909 (97.7) | 113 (2.3) | 1 |  |  |  |
| Prior PCI, n (%) |  |  |  |  |  |  |
| Yes | 2,043 (97.6) | 50 (2.4) | 0.9 (0.6, 1.3) | 0.648 |  |  |
| No | 2,941 (97.8) | 66 (2.2) | 1 |  |  |  |
| Prior CABG, n (%) |  |  |  |  |  |  |
| Yes | 55 (94.8) | 3 (5.2) | 0.4 (0.1, 1.4) | 0.149 |  |  |
| No | 4,929 (97.8) | 113 (2.2) | 1 |  |  |  |
| Prior MI, n (%) |  |  |  |  |  |  |
| Yes | 1,569 (97.8) | 36 (2.2) | 1.0 (0.7, 1.5) | 0.919 |  |  |
| No | 3,415 (97.7) | 80 (2.3) | 1 |  |  |  |
| Cardiogenic shock at start of PCI, n (%) |  |  |  |  |  |  |
| Yes | 38 (97.4) | 1 (2.6) | 0.9 (0.1, 6.5) | 0.903 |  |  |
| No | 4,946 (97.7) | 115 (2.3) | 1 |  |  |  |
| CAD presentation, n (%) |  |  |  |  |  |  |
| STEMI | 145 (98.0) | 3 (2.0) | 1.1 (0.3, 3.5) | 0.889 |  |  |
| NSTEMI / Unstable Angina | 1,370 (97.5) | 35 (2.5) | 0.9 (0.6, 1.3) | 0.535 |  |  |
| Stable CAD | 3,469 (97.8) | 78 (2.2) | 1 |  |  |  |
| LVEF, %, mean (SD) | 55.7 (15.2) | 48.7 (17.5) | 1.03 (1.01, 1.04) | <0.001 |  |  |
| Bifurcation lesion, n (%) |  |  |  |  |  |  |
| Yes | 598 (98.5) | 9 (1.5) | 1.6 (0.8, 3.2) | 0.162 |  |  |
| No | 4,358 (97.6) | 107 (2.4) | 1 |  |  |  |
| Previously treated lesion, n (%) |  |  |  |  |  |  |
| Yes | 181 (95.3) | 9 (4.7) | 0.4 (0.2, 0.9) | 0.024 |  |  |
| No | 4,790 (97.8) | 107 (2.2) | 1 |  |  |  |
| Initial access site, n (%) |  |  |  |  |  |  |
| Femoral only | 2,532 (97.5) | 65 (2.5) | 0.8 (0.5, 1.1) | 0.198 |  |  |
| Combination | 49 (94.2) | 3 (5.8) | 0.3 (0.1, 1.1) | 0.068 |  |  |
| Radial only | 2,395 (98.0) | 48 (2.0) | 1 |  |  |  |
| Plaque modification, n (%) |  |  |  |  |  |  |
| Yes | 223 (99.1) | 2 (0.9) | 2.6 (0.6, 10.6) | 0.181 |  |  |
| No | 4,741 (97.7) | 111 (2.3) | 1 |  |  |  |

Table S6. Factors associated with complications in patients who did not meet IVUS criteria

| Characteristics | Complications | | Univariate | | Multivariate | |
| --- | --- | --- | --- | --- | --- | --- |
|  | Yes | No | OR (95% CI) | P value | OR (95% CI) | P value |
|  | n = 161 | n = 4,939 |  |  |  |  |
| Indication-IVUS, n (%) |  |  |  |  |  |  |
| C-unmatched-IVUS | 38 (6.6) | 540 (93.4) | 2.5 (1.7, 3.7) | <0.001 | 1.9 (1.3, 2.8) | 0.001 |
| C-unmatched-nonIVUS | 123 (2.7) | 4,399 (97.3) | 1 |  | 1 |  |
| Prior MI, n (%) |  |  |  |  |  |  |
| Yes | 65 (4.0) | 1,540 (96.0) | 1.5 (1.1, 2.1) | 0.014 | 1.5 (1.1, 2.0) | 0.024 |
| No | 96 (2.7) | 3,399 (97.3) | 1 |  | 1 |  |
| Fluoroscopy time, minutes, median (range) | 18.5 (0.8, 162.4) | 11.1 (0.5, 910.0) | 1.015 (1.008, 1.022) | <0.001 | 1.005 (1.001, 1.010) | 0.017 |
| Total volume of contrast, ml, median (range) | 120.0 (40.0, 600.0) | 100.0 (20.0, 470.0) | 1.008 (1.005, 1.010) | <0.001 | 1.006 (1.004, 1.009) | <0.001 |
| Gender, n (%) |  |  |  |  |  |  |
| Female | 56 (3.8) | 1,407 (96.2) | 1.3 (1.0, 1.9) | 0.083 |  |  |
| Male | 105 (2.9) | 3,532 (97.1) | 1 |  |  |  |
| Age, years, mean (SD) | 62.8 (10.0) | 62.3 (10.7) | 1.00 (0.99, 1.02) | 0.556 |  |  |
| Diabetes mellitus, n (%) |  |  |  |  |  |  |
| Yes | 57 (3.1) | 1,781 (96.9) | 1.0 (0.7, 1.3) | 0.864 |  |  |
| No | 104 (3.2) | 3,158 (96.8) | 1 |  |  |  |
| Hypertension, n (%) |  |  |  |  |  |  |
| Yes | 106 (3.0) | 3,463 (97.0) | 0.8 (0.6, 1.1) | 0.245 |  |  |
| No | 55 (3.6) | 1,476 (96.4) | 1 |  |  |  |
| Dyslipidemia, n (%) |  |  |  |  |  |  |
| Yes | 120 (3.2) | 3,603 (96.8) | 1.1 (0.8, 1.6) | 0.656 |  |  |
| No | 41 (3.0) | 1,336 (97.0) | 1 |  |  |  |
| Peripheral Arterial Disease, n (%) |  |  |  |  |  |  |
| Yes | 5 (6.4) | 73 (93.6) | 2.1 (0.9, 5.4) | 0.106 |  |  |
| No | 156 (3.1) | 4,866 (96.9) | 1 |  |  |  |
| Prior PCI, n (%) |  |  |  |  |  |  |
| Yes | 78 (3.7) | 2,015 (96.3) | 1.4 (1.0, 1.9) | 0.053 |  |  |
| No | 83 (2.8) | 2,924 (97.2) | 1 |  |  |  |
| Prior CABG, n (%) |  |  |  |  |  |  |
| Yes | 3 (5.2) | 55 (94.8) | 1.7 (0.5, 5.4) | 0.383 |  |  |
| No | 158 (3.1) | 4,884 (96.9) | 1 |  |  |  |
| Cardiogenic shock at start of PCI, n (%) |  |  |  |  |  |  |
| Yes | 3 (7.7) | 36 (92.3) | 2.6 (0.8, 8.5) | 0.117 |  |  |
| No | 158 (3.1) | 4,903 (96.9) | 1 |  |  |  |
| CAD presentation, n (%) |  |  |  |  |  |  |
| STEMI | 5 (3.4) | 143 (96.6) | 1.0 (0.4, 2.4) | 0.954 |  |  |
| NSTEMI / Unstable Angina | 33 (2.3) | 1,372 (97.7) | 0.7 (0.5, 1.0) | 0.043 |  |  |
| Stable CAD | 123 (3.5) | 3,424 (96.5) | 1 |  |  |  |
| LVEF, %, mean (SD) | 53.2 (15.5) | 55.6 (15.3) | 0.99 (0.98, 1.00) | 0.090 |  |  |
| Lesion complexity, n (%) |  |  |  |  |  |  |
| B2 or C | 126 (3.6) | 3,363 (96.4) | 1.7 (1.2, 2.5) | 0.007 |  |  |
| A or B1 | 34 (2.2) | 1,543 (97.8) | 1 |  |  |  |
| Bifurcation lesion, n (%) |  |  |  |  |  |  |
| Yes | 30 (4.9) | 577 (95.1) | 1.7 (1.2, 2.6) | 0.007 |  |  |
| No | 129 (2.9) | 4,336 (97.1) | 1 |  |  |  |
| Previously treated lesion, n (%) |  |  |  |  |  |  |
| Yes | 10 (5.3) | 180 (94.7) | 1.8 (0.9, 3.4) | 0.089 |  |  |
| No | 149 (3.0) | 4,748 (97.0) | 1 |  |  |  |
| Initial access site, n (%) |  |  |  |  |  |  |
| Femoral only | 91 (3.5) | 2,506 (96.5) | 1.3 (1.0, 1.8) | 0.085 |  |  |
| Combination | 5 (9.6) | 47 (90.4) | 3.9 (1.5, 10.1) | 0.005 |  |  |
| Radial only | 65 (2.7) | 2,378 (97.3) | 1 |  |  |  |
| Plaque modification, n (%) |  |  |  |  |  |  |
| Yes | 11 (4.9) | 214 (95.1) | 1.6 (0.9, 3.1) | 0.125 |  |  |
| No | 148 (3.1) | 4,704 (96.9) | 1 |  |  |  |

Table S7. Factors associated with in hospital death in patients who did not meet IVUS criteria

| Characteristics | Death | | Univariate | | Multivariate | |
| --- | --- | --- | --- | --- | --- | --- |
|  | Yes | No | OR (95% CI) | P value | OR (95% CI) | P value |
|  | n = 9 | n = 5,091 |  |  |  |  |
| Indication-IVUS, n (%) |  |  |  |  |  |  |
| C-unmatched-IVUS | 2 (0.3) | 576 (99.7) | 2.2 (0.5, 10.8) | 0.315 | 3.0 (0.5, 16.4) | 0.215 |
| C-unmatched-nonIVUS | 7 (0.2) | 4,515 (99.8) | 1 |  | 1 |  |
| Cardiogenic shock at start of PCI, n (%) |  |  |  |  |  |  |
| Yes | 3 (7.7) | 36 (92.3) | 70.2 (16.9, 291.7) | <0.001 | 52.0 (10.3, 261.8) | <0.001 |
| No | 6 (0.1) | 5,055 (99.9) | 1 |  | 1 |  |
| Peripheral Arterial Disease, n (%) |  |  |  |  |  |  |
| Yes | 3 (3.8) | 75 (96.2) | 33.4 (8.2, 136.2) | <0.001 | 13.9 (2.7, 71.4) | 0.002 |
| No | 6 (0.1) | 5,016 (99.9) | 1 |  | 1 |  |
| Age, years, mean (SD) | 74.0 (12.2) | 62.3 (10.7) | 1.1 (1.0, 1.2) | 0.001 | 1.1 (1.0, 1.2) | 0.010 |
| CAD presentation, n (%) |  |  |  |  |  |  |
| ACS | 4 (0.3) | 1,549 (99.7) | 1.8 (0.5, 6.8) | 0.368 |  |  |
| Stable CAD | 5 (0.1) | 3,542 (99.9) | 1 |  |  |  |
| Femoral access, n (%) |  |  |  |  |  |  |
| Yes | 8 (0.3) | 2,679 (99.7) | 7.2 (0.9, 57.6) | 0.063 |  |  |
| No | 1 (0.04) | 2,412 (99.96) | 1 |  |  |  |
| Gender, n (%) |  |  |  |  |  |  |
| Female | 5 (0.3) | 1,458 (99.7) | 3.1 (0.8, 11.6) | 0.091 |  |  |
| Male | 4 (0.1) | 3,633 (99.9) | 1 |  |  |  |
| Diabetes mellitus, n (%) |  |  |  |  |  |  |
| Yes | 3 (0.2) | 1,835 (99.8) | 0.9 (0.2, 3.6) | 0.866 |  |  |
| No | 6 (0.2) | 3,256 (99.8) | 1 |  |  |  |
| Hypertension, n (%) |  |  |  |  |  |  |
| Yes | 6 (0.2) | 3,563 (99.8) | 0.9 (0.2, 3.4) | 0.828 |  |  |
| No | 3 (0.2) | 1,528 (99.8) | 1 |  |  |  |
| Dyslipidemia, n (%) |  |  |  |  |  |  |
| Yes | 4 (0.1) | 3,719 (99.9) | 0.3 (0.1, 1.1) | 0.069 |  |  |
| No | 5 (0.4) | 1,372 (99.6) | 1 |  |  |  |
| Prior PCI, n (%) |  |  |  |  |  |  |
| Yes | 1 (0.0) | 2,092 (100.0) | 0.2 (0.0, 1.4) | 0.105 |  |  |
| No | 8 (0.3) | 2,999 (99.7) | 1 |  |  |  |
| Prior MI, n (%) |  |  |  |  |  |  |
| Yes | 1 (0.1) | 1,604 (99.9) | 0.3 (0.0, 2.2) | 0.219 |  |  |
| No | 8 (0.2) | 3,487 (99.8) | 1 |  |  |  |
| LVEF, %, mean (SD) | 45.8 (24.4) | 55.5 (15.3) | 0.96 (0.91, 1.02) | 0.215 |  |  |
| Lesion complexity, n (%) |  |  |  |  |  |  |
| B2 or C | 6 (0.2) | 3,483 (99.8) | 0.9 (0.2, 3.6) | 0.886 |  |  |
| A or B1 | 3 (0.2) | 1,574 (99.8) | 1 |  |  |  |
| Plaque modification, n (%) |  |  |  |  |  |  |
| Yes | 1 (0.4) | 224 (99.6) | 2.7 (0.3, 21.7) | 0.349 |  |  |
| No | 8 (0.2) | 4,844 (99.8) | 1 |  |  |  |
| Fluoroscopy time, minutes, median (range) | 19.7 (5.9, 71.1) | 11.2 (0.5, 910.0) | 1.00 (0.99, 1.01) | 0.395 |  |  |
| Total volume of contrast, ml, median (range) | 90.0 (50.0, 170.0) | 100.0 (20.0, 600.0) | 1.00 (0.99, 1.01) | 0.873 |  |  |

Table S8. Factors associated with death in 1 year in patients who did not meet IVUS criteria

| Characteristics | Death in 1 year | | Univariate | | Multivariate | |
| --- | --- | --- | --- | --- | --- | --- |
|  | Yes | No | OR (95% CI) | P value | OR (95% CI) | P value |
|  | n = 180 | n = 4,920 |  |  |  |  |
| Indication-IVUS, n (%) |  |  |  |  |  |  |
| C-unmatched-IVUS | 21 (3.6) | 557 (96.4) | 1.0 (0.7, 1.6) | 0.886 | 1.1 (0.7, 1.8) | 0.722 |
| C-unmatched-nonIVUS | 159 (3.5) | 4,363 (96.5) | 1 |  | 1 |  |
| Age, years, mean (SD) | 69.0 (11.2) | 62.1 (10.6) | 1.07 (1.05, 1.08) | <0.001 | 1.06 (1.05, 1.08) | <0.001 |
| Peripheral Arterial Disease, n (%) |  |  |  |  |  |  |
| Yes | 12 (15.4) | 66 (84.6) | 5.3 (2.8, 9.9) | <0.001 | 4.2 (2.1, 8.2) | <0.001 |
| No | 168 (3.3) | 4,854 (96.7) | 1 |  | 1 |  |
| Dyslipidemia, n (%) |  |  |  |  |  |  |
| Yes | 113 (3.0) | 3,610 (97.0) | 0.6 (0.4, 0.8) | 0.002 | 0.6 (0.4, 0.8) | 0.003 |
| No | 67 (4.9) | 1,310 (95.1) | 1 |  | 1 |  |
| Cardiogenic shock at start of PCI, n (%) |  |  |  |  |  |  |
| Yes | 6 (15.4) | 33 (84.6) | 5.1 (2.1, 12.3) | <0.001 | 3.4 (1.3, 8.7) | 0.010 |
| No | 174 (3.4) | 4,887 (96.6) | 1 |  | 1 |  |
| CAD presentation, n (%) |  |  |  |  |  |  |
| ACS | 66 (4.2) | 1,487 (95.8) | 1.3 (1.0, 1.8) | 0.066 | 1.4 (1.0, 1.9) | 0.049 |
| Stable CAD | 114 (3.2) | 3,433 (96.8) | 1 |  | 1 |  |
| Gender, n (%) |  |  |  |  |  |  |
| Female | 61 (4.2) | 1,402 (95.8) | 1.3 (0.9, 1.8) | 0.117 |  |  |
| Male | 119 (3.3) | 3,518 (96.7) | 1 |  |  |  |
| Diabetes mellitus, n (%) |  |  |  |  |  |  |
| Yes | 74 (4.0) | 1,764 (96.0) | 1.2 (0.9, 1.7) | 0.150 |  |  |
| No | 106 (3.2) | 3,156 (96.8) | 1 |  |  |  |
| Hypertension, n (%) |  |  |  |  |  |  |
| Yes | 135 (3.8) | 3,434 (96.2) | 1.3 (0.9, 1.8) | 0.136 |  |  |
| No | 45 (2.9) | 1,486 (97.1) | 1 |  |  |  |
| Prior PCI, n (%) |  |  |  |  |  |  |
| Yes | 64 (3.1) | 2,029 (96.9) | 0.8 (0.6, 1.1) | 0.129 |  |  |
| No | 116 (3.9) | 2,891 (96.1) | 1 |  |  |  |
| Prior CABG, n (%) |  |  |  |  |  |  |
| Yes | 1 (1.7) | 57 (98.3) | 0.5 (0.1, 3.5) | 0.464 |  |  |
| No | 179 (3.6) | 4,863 (96.4) | 1 |  |  |  |
| Prior MI, n (%) |  |  |  |  |  |  |
| Yes | 43 (2.7) | 1,562 (97.3) | 0.7 (0.5, 1.0) | 0.027 |  |  |
| No | 137 (3.9) | 3,358 (96.1) | 1 |  |  |  |
| Lesion complexity, n (%) |  |  |  |  |  |  |
| B2 or C | 134 (3.8) | 3,355 (96.2) | 1.4 (1.0, 1.9) | 0.079 |  |  |
| A or B1 | 45 (2.9) | 1,532 (97.1) | 1 |  |  |  |
| LVEF, %, mean (SD) | 44.6 (17.2) | 55.9 (15.1) | 0.96 (0.95, 0.97) | <0.001 |  |  |
| Bifurcation lesion, n (%) |  |  |  |  |  |  |
| Yes | 19 (3.1) | 588 (96.9) | 0.9 (0.5, 1.4) | 0.553 |  |  |
| No | 161 (3.6) | 4,304 (96.4) | 1 |  |  |  |
| Previously treated lesion, n (%) |  |  |  |  |  |  |
| Yes | 5 (2.6) | 185 (97.4) | 0.7 (0.3, 1.8) | 0.492 |  |  |
| No | 175 (3.6) | 4,722 (96.4) | 1 |  |  |  |
| Femoral access, n (%) |  |  |  |  |  |  |
| Yes | 98 (3.6) | 2,589 (96.4) | 1.1 (0.8, 1.5) | 0.631 |  |  |
| No | 82 (3.4) | 2,331 (96.6) | 1 |  |  |  |
| Plaque modification, n (%) |  |  |  |  |  |  |
| Yes | 11 (4.9) | 214 (95.1) | 1.4 (0.8, 2.7) | 0.259 |  |  |
| No | 168 (3.5) | 4,684 (96.5) | 1 |  |  |  |
| Fluoroscopy time, minutes, median (range) | 11.2 (1.4, 143.2) | 11.2 (0.5, 910.0) | 1.002 (0.998, 1.007) | 0.357 |  |  |
| Total volume of contrast, ml, median (range) | 100.0 (30.0, 250,0) | 100.0 (20.0, 600.0) | 0.998 (0.995, 1.002) | 0.336 |  |  |

Table S9 Balance of factors associated with IVUS-guided applications

| Estimation | Met IVUS criteria (C^+^group) | | | | Unmet IVUS criteria (C^-^group) | | | |
| --- | --- | --- | --- | --- | --- | --- | --- | --- |
|  | Standardized differences | | Variance ratio | | Standardized differences | | Variance ratio | |
|  | Raw | Weighted | Raw | Weighted | Raw | Weighted | Raw | Weighted |
| Total volume of contrast, ml | 0.594 | -0.014 | 1.942 | 0.633 | 0.637 | 0.128 | 1.353 | 0.634 |
| Plaque modification vs None | 0.470 | -0.008 | 4.945 | 0.966 | 0.480 | 0.013 | 4.984 | 1.060 |
| Bifurcation lesion vs None | 0.478 | -0.005 | 2.110 | 0.989 | 0.351 | 0.009 | 1.914 | 1.022 |
| CAD presentation ACS vs Stable CAD | -0.507 | 0.004 | 1.184 | 0.996 | -0.260 | 0.009 | 0.751 | 1.007 |
| Chronic total occlusion vs None | 0.325 | 0.028 | 1.893 | 1.064 | - | - | - | - |
| Prior MI vs None | 0.279 | 0.009 | 1.397 | 1.013 | - | - | - | - |
| Age, years | -0.058 | -0.040 | 1.007 | 1.089 | - | - | - | - |
| Previously treated lesion vs None | 0.220 | 0.015 | 2.181 | 1.064 | - | - | - | - |
| CKD vs None | -0.063 | 0.006 | 0.977 | 1.002 | - | - | - | - |
| Prior PCI vs None | 0.389 | 0.011 | 1.405 | 1.012 | - | - | - | - |
| Peripheral Arterial Disease vs None | 0.085 | 0.013 | 1.828 | 1.104 | - | - | - | - |
| Femoral access | - | - | - | - | 0.369 | -0.031 | 0.862 | 1.002 |
| Lesion complexity B2 or C vs A or B1 | - | - | - | - | 0.291 | 0.010 | 0.725 | 0.991 |

**Figure S1.** Balance plot of kernel density of each covariate by IVUS-guided PCI (I+) vs angio-guided PCI (I-) stratify by meet and unmeet IVUS criteria.

1. IVUS met criteria (C+)


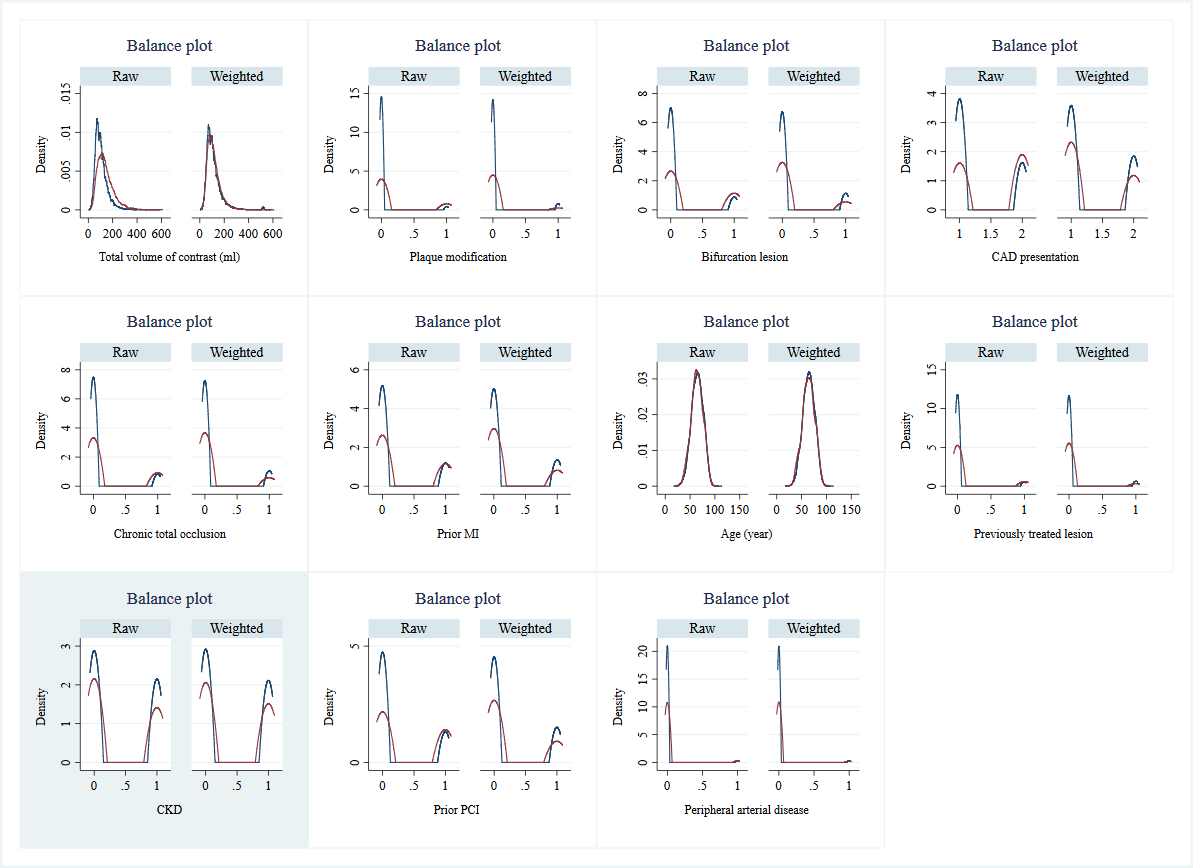


1. IVUS unmet criteria (C-)


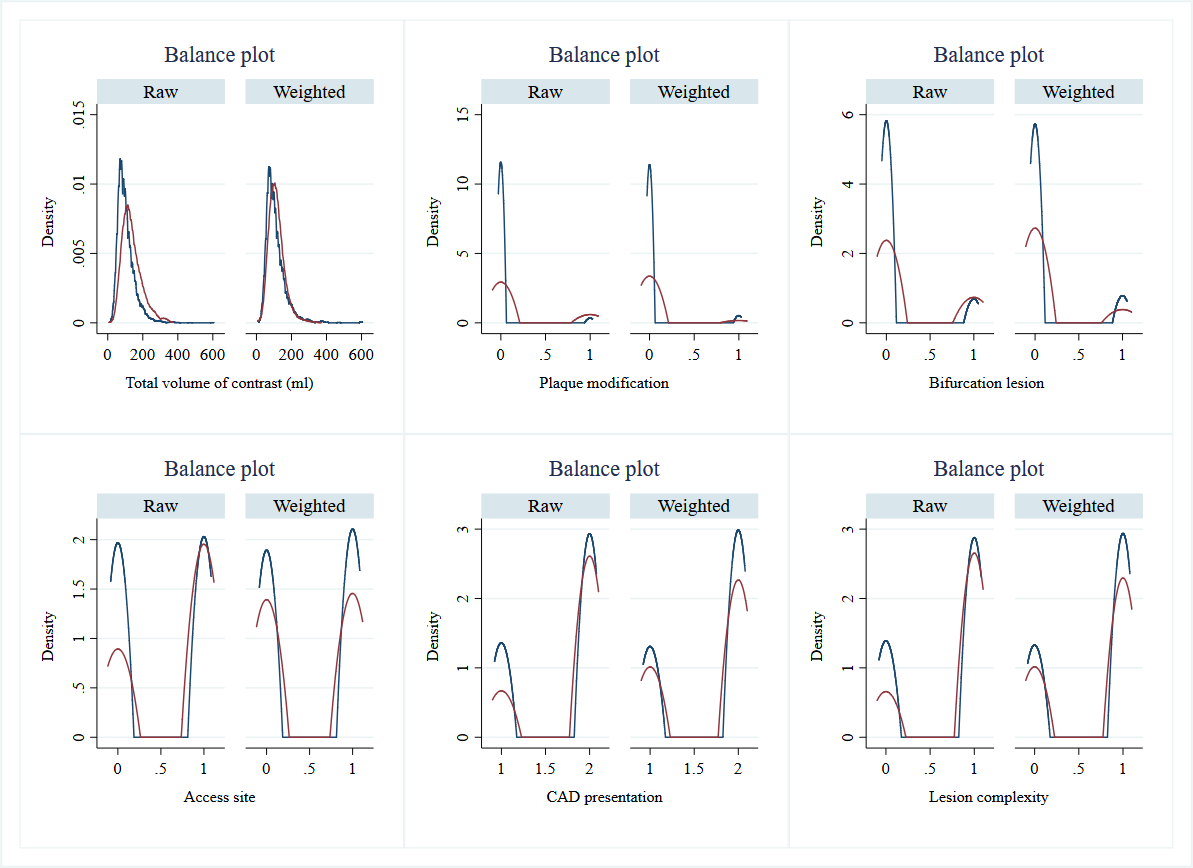


**Figure S2.** Overlapping plot: Estimating densities of the probability of IVUS-guided PCI (I+) and angio-guided PCI (I-) to assess if each individual patient has a positive probability of applying and not applying IVUS.

1. IVUS met criteria


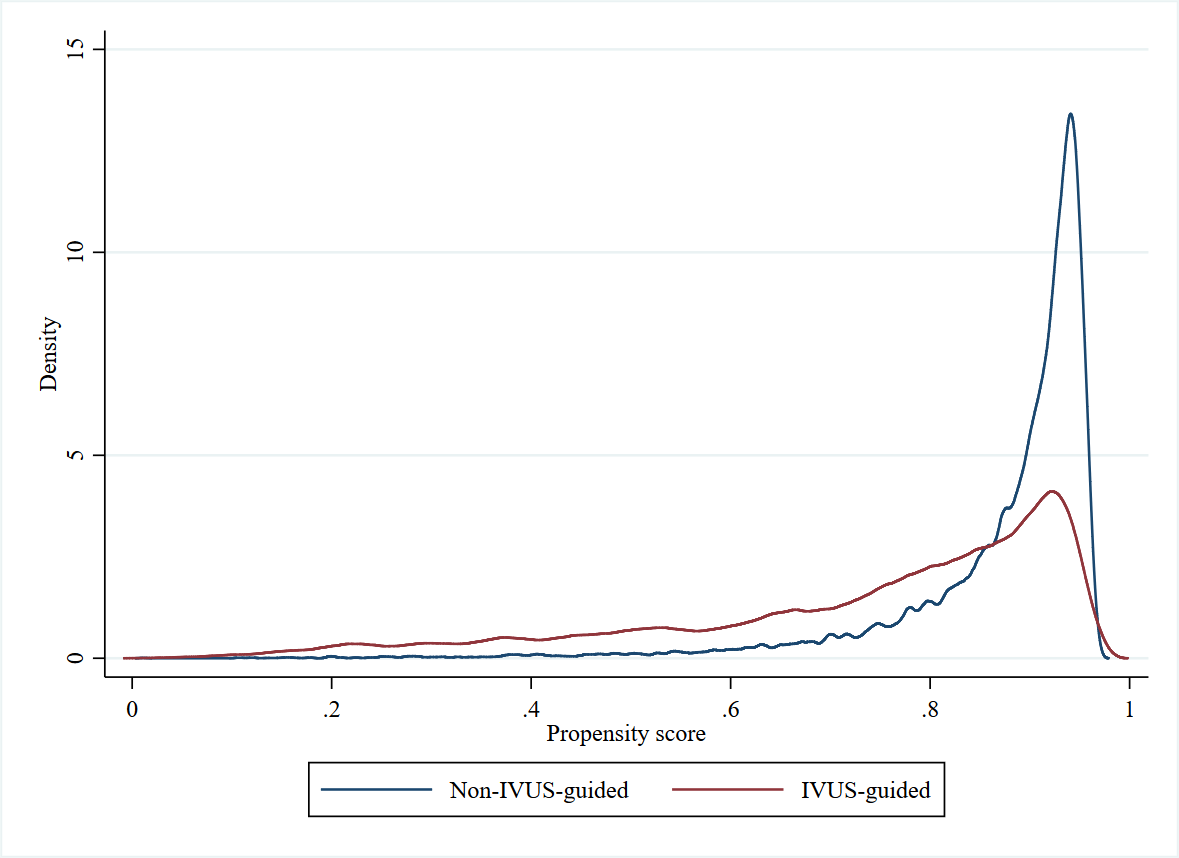


1. IVUS unmet criteria


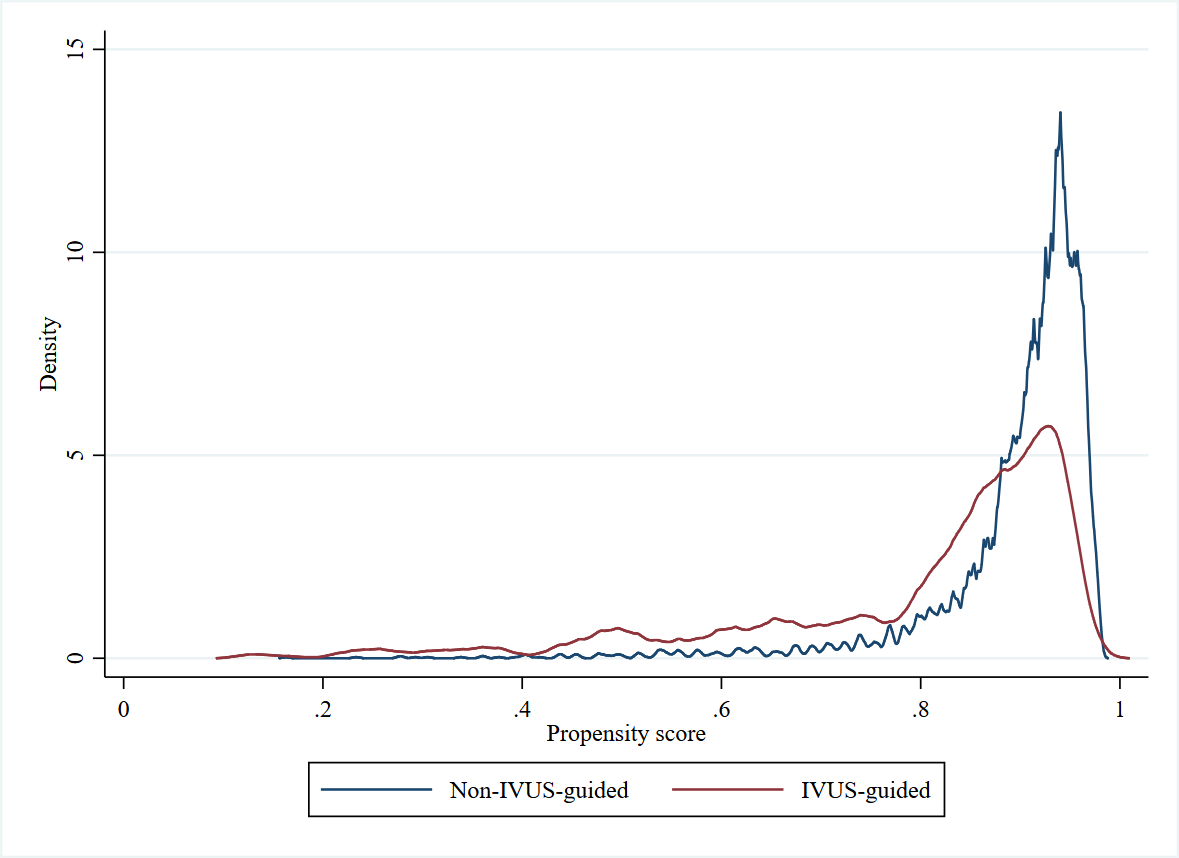

Supplement: Supplementary file 1 — Supplementary Information. [file 41598_2022_27250_MOESM1_ESM.docx]
